# Visualizing Trimming Dependence of Biodistribution and Kinetics with Homo- and Heterogeneous *N*-Glycoclusters on Fluorescent Albumin

**DOI:** 10.1038/srep21797

**Published:** 2016-02-23

**Authors:** Akihiro Ogura, Tsuyoshi Tahara, Satoshi Nozaki, Koji Morimoto, Yasuhiko Kizuka, Shinobu Kitazume, Mitsuko Hara, Soichi Kojima, Hirotaka Onoe, Almira Kurbangalieva, Naoyuki Taniguchi, Yasuyoshi Watanabe, Katsunori Tanaka

**Affiliations:** 1 Biofunctional Synthetic Chemistry Laboratory, RIKEN, 2-1 Hirosawa, Wako-shi, Saitama 351-0198, Japan; 2 RIKEN Center for Life Science Technologies, 6-7-3 Minatojima-minamimachi, Chuo-ku, Kobe, Hyogo 650-0047, Japan; 3 Osaka Women’s Junior College, 3-8-1 Kasugaoka, Fujiidera-shi, Osaka, 583-8558, Japan; 4 Disease Glycomics Team, Global Research Cluster, RIKEN-Max Planck Joint Research Center for Systems Chemical Biology, RIKEN, 2-1 Hirosawa, Wako-shi, Saitama 351-0198, Japan; 5 Micro-Signaling Regulation Technology Unit, RIKEN Center for Life Science Technologies, Wako-shi, Saitama, 351-0198, Japan; 6 Biofunctional Chemistry Laboratory, A. Butlerov Institute of Chemistry, Kazan Federal University, 18 Kremlyovskaya Street, Kazan 420008, Russia; 7 Japan Science and Technology Agency-PRESTO, 2-1 Hirosawa, Wako-shi, Saitama 351-0198, Japan

## Abstract

A series of *N*-glycans, each sequentially trimmed from biantennary sialoglycans, were homo- or heterogeneously clustered efficiently on fluorescent albumin using a method that combined strain-promoted alkyne-azide cyclization and 6π-azaelectrocyclization. Noninvasive *in vivo* kinetics and dissection analysis revealed, for the first time, a glycan-dependent shift from urinary to gall bladder excretion mediated by sequential trimming of non-reducing end sialic acids. *N*-glycoalbumins that were trimmed further, in particular, GlcNAc- and hybrid biantennary-terminated congeners, were selectively taken up by sinusoidal endothelial and stellate cells in the liver, which are critical for diagnosis and treatment of liver fibrillation. Our glycocluster strategy can not only reveal the previously unexplored extracellular functions of *N*-glycan trimming, but will be classified as the newly emerging glycoprobes for diagnostic and therapeutic applications.

Recent advances in glycochemical biology and analytical techniques greatly facilitated the understanding of the glycan functions^1^. In particular, *N*-glycans, which are bound to asparagine residues, intracellularly act as molecular signals for protein transport, sensors for the folding or degradation of misfolded proteins (Fig. 1)^2^. On the other hand, the precise molecular recognition mechanisms of the variously trimmed extracellular *N*-glycans, e.g., those on soluble proteins and cell surfaces, remain largely unknown^3^. For example, the sialic acids at the non-reducing end of *N*-glycans contribute to the circulatory residence of glycoproteins^4^,^5^,^6^; however, unlike nucleic acids or proteins, these glycans are not necessarily uniform in structure. In addition, various glycan structures form homogeneous or heterogeneous clusters on proteins or cells, creating challenges for analysis of recognition by both conventional and synthetic methods. It has been postulated that glycan structural diversity could lead to selective interactions in a variety of important physiological events, including cell recognition, adhesion, and signal transduction, through a pattern-recognition mechanism (Fig. 1)^7^,^8^,^9^. Therefore, mimicking glycan pattern recognition by reconstructing homogeneous and heterogeneous glycoclusters could not only facilitate understanding of the trimming-dependent interaction mechanisms and functions of *N*-glycans on proteins or cells, but could also provide a strategy for development of innovative diagnostic and therapeutic *N*-glycoconjugate tracers.

Among a number of glycoconjugates or clusters reported on dendrimers, liposomes, nanoparticles, and protein templates for biodistribution studies^10^,^11^,^12^,^13^,^14^,^15^,^16^,^17^,^18^,^19^, systematic studies have been performed using human serum albumin (HSA), which is conjugated with mono-saccharides that mimic the non-reducing ends of whole glycans^20^. HSA has several advantages as a cluster template: (i) availability of the more than 30 lysines for glycan clusterization, (ii) serum stability, and (iii) negligible immunogenicity in clinical applications. In fact, the albumin conjugates can direct monosaccharide-dependent clearance and organ-selective accumulation; ^99m^Tc-galactose–clustered HSA has already been clinically approved for estimation of hepatocyte mass and function^21^.

On the other hand, only a few reports have addressed glycoclusters containing whole *N*-glycan structures. This is due to limitations on the availability of *N*-glycans by either isolation or synthesis, as well as the difficulty of derivatizing and/or clustering the complex and hydrophilic *N*-glycan molecules using currently available conjugation methods. André, Gabius, and Unverzagt have succeeded in conjugating albumin with various di-, tri-, and tetra-antennary *N*-glycans, which were efficiently prepared by their own chemoenzymatic methods, despite a few molecules per one albumin, through the isothiocyanate ligation^17^,^18^,^19^. However, systematic mouse studies using these ^125^l-labeled neo-glycoproteins did not yield clear-cut conclusions about glycan-dependent *in vivo* kinetics and biodistribution, presumably due to the inefficient *N*-glycan valency, i.e., the absence of multiple glycans on each molecule of albumin, to effect the cluster effects. Our group recently reported the synthesis of polylysine-based glycodendrimers with 16 molecules of biantennary *N*-glycans via the Cu-mediated Huisgen cycloaddition reaction, and we have successfully mimicked the cluster effects of *N*-glycans and visualized the sialic acid-dependent circulatory residence by Positron Emission Tomography (PET)^10^. However, the problems associated with serum instability of unnatural dendrimers, as well as the incompatibility of existing synthesis methods with diversification of the *N*-glycocluster structures, hampered further studies of detailed *N*-glycan–dependent biodistribution and accumulation mechanisms at the cellular and protein levels.

Taking into account the results of previous reports and the aforementioned advantages of albumin, we postulated that efficient chemical conjugation of various *N*-glycans to produce *N*-glycoclusters on albumin could facilitate analysis of the trimming dependence of biodistribution and kinetics. In addition to a number of reports describing “bioorthogonal” bioconjugation, in which glycans were conjugated to engineered proteins via reactions such as the Cu(I)-catalyzed Huisgen cycloaddition and its variants^22^,^23^, we have independently developed 6π-azaelectrocyclization of unsaturated imines as an efficient method for directly modifying native lysines^24^,^25^. This reaction proceeds selectively with lysine amino groups under aqueous conditions at ambient temperatures in almost quantitative yields, and is much more reactive (1,000 times faster) and therefore more efficient than other conventional methods such as *N*-hydroxysuccinimide or isothiocyanate reagents. It is worth mentioning that the number of molecules conjugated to the lysines can precisely be controlled by adjusting the probe and protein concentrations, allowing two different molecules to be introduced onto proteins in a precise ratio. Therefore, we applied our azaelectrocyclization in combination with the strain-promoted click reaction^26^ to efficiently conjugate various *N*-glycoclusters on albumin (Fig. 2a). Based on established electrocyclization protocols, not only homogeneous glycoclusters containing about ten molecules of complex-type *N*-glycans, but also heterogeneous clusters with a precisely defined ratio of two different glycan molecules, could be readily prepared. Noninvasive *in vivo* kinetics, distribution, and fluorescence microscope analyses of the fluorescently labeled *N*-glycoalbumins allowed, for the first time, clear visualization of the effects of *N*-glycan trimming, which plays a critical role in controlling biodistribution and metabolic pathways. These effects are distinct from those of the monosaccharide-modified albumins used previously as whole-glycan mimics.

## Experiments and Results

**Figure 2:**
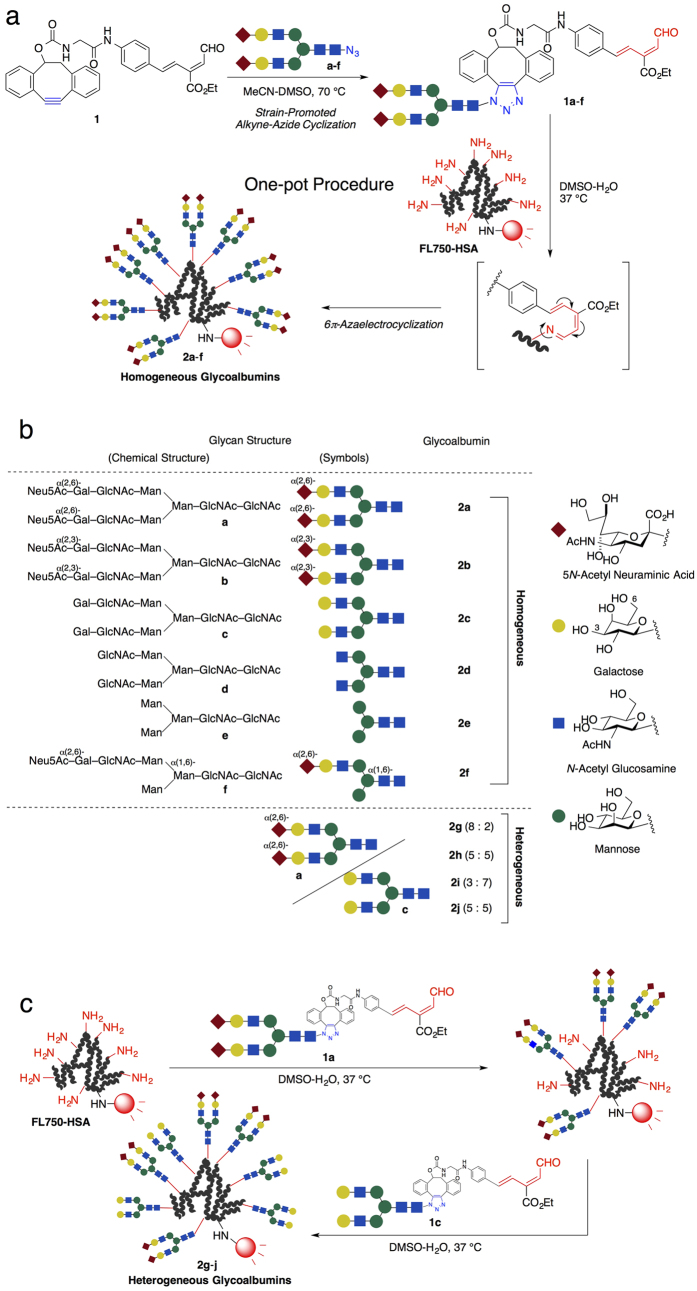
Preparation of homo- and heterogeneous *N*-glycoalbumins through a combination of the strain-promoted click reaction and 6π-azaelectrocyclization. (**a**) Azide-functionalized glycans **a**–**f** (see Supplementary Information for details) were initially treated with **1** at 70 °C in MeCN-DMSO, and **1a–f** generated *in situ* were then conjugated to HiLyte Fluor 750-labeled HSA (FL750-HSA) at 37 °C in DMSO-water. A simple ultrafiltration procedure afforded the homogeneous *N*-glycoalbumins **2a–f**. (**b**) List and structures of homo- and heterogeneous *N*-glycoalbumins. Approximately 10 molecules of glycans were introduced to albumin, as revealed by MALDI-TOF mass spectroscopy analysis. Numbers in parentheses for heterogeneous clusters **2g–j** represent the ratios and order of the introduced glycans. (**c**) Preparation of heterogeneous *N*-glycoalbumins.

We previously reported two-step and one-pot glycan conjugation to proteins via a method that combined the Staudinger reaction and azaelectrocyclization^27^. However, glycan-phosphine derivatives are readily oxidized, preventing this method from being applied to a wide range of conjugating molecules. To address this issue, we improved the method by substituting the Staudinger reaction with the strain-promoted click reaction^26^, which turned out to be generally applicable to a wide range of *N*-glycan derivatives investigated in this research. As shown in Fig. 2, various azide-terminated glycans **a**-**f**^10^,^28^ were initially treated with freshly prepared cyclooctyne-aldehyde **1** 29 (details in Supplementary Information). Heating the mixture speeded up quantitative completion of the strain-assisted click reaction, which could be conveniently monitored by HPLC (Supplementary Information). To this mixture was added an aqueous solution of the HiLyte Fluor 750^®^-labeled HSA (**FL750-HSA**, preparation and properties in Supplementary Information), and 6π-azaelectrocyclization was performed at 37 °C to conjugate glycans to albumin. After small molecules were simply filtered off by Amicon^®^ centrifugation, *N*-glycan conjugation was evaluated by MALDI-TOF mass spectroscopy. Due to the high reactivity of azaelectrocyclization in comparison with the previously used isothiocyanate ligation, about a dozen glycan molecules were introduced into each molecule of **FL750-HSA**, independent of the *N*-glycan structures (Fig. 2b, Supplementary Information). It should be noted that each trimmed *N*-glycocluster **2b**–**f** from the disialoglycocluster **2a** could not be easily accessed by sequential trimming with site-selective glycosidases (e.g., sialidase treatment of **2a** to prepare **2c**), because the incomplete enzymatic reaction could produce uncontrolled heterogeneous *N*-glycoclusters, and it is not necessarily feasible to monitor the reaction process and confirm and isolate the products (*vide infra*).

High reactivity could also precisely control the number of the conjugating *N*-glycans by adjusting the amount of the probes **1a**–**f**; hence, albumin conjugates with a few molecules of *N*-glycans, which were used as a control sample to evaluate the importance of glycocluster valency, could be easily prepared (Supplementary Information). The conjugation-control properties of the new method highlights the construction of glycan structure–defined heterogeneous clusters on albumin, which is otherwise difficult to achieve by previous methods including glycosidase-catalyzed trimming (Fig. 2c). In particular, we focused on heterogeneous glycoalbumins containing different ratios of two-types of sialo- and asialoglycans **a** and **c**. Thus, **FL750-HSA** was initially reacted with 17.5 eq of the aldehyde **1a** to give the glycoconjugate with eight molecules of the sialo-*N*-glycan. The intermediate glycoalbumin was then treated with 7.5 eq of aldehyde **1c** to introduce an additional two molecules of asialo-*N*-glycan, affording the heterogeneous glycoalbumin **2g** (introduced glycans **a** : **c** = 8 : 2). We also introduced a different ratio of sialo- and asialoglycans on **2h** (**a** : **c** = 5 : 5) and **2i** (**a** : **c** = 3 : 7) by simply modifying the equivalents of the electrocyclization probes (see details in Supplementary Information). In order to check the positional effects of the glycans introduced on lysines of **FL750-HSA**, inversely treated glycoalbumin **2j** with sialo- and asialoglycan-derived aldehydes (first by **1c**, and then by **1a**), was also prepared by similar procedure (**a** : **c** = 5 : 5).

**Figure 3:**
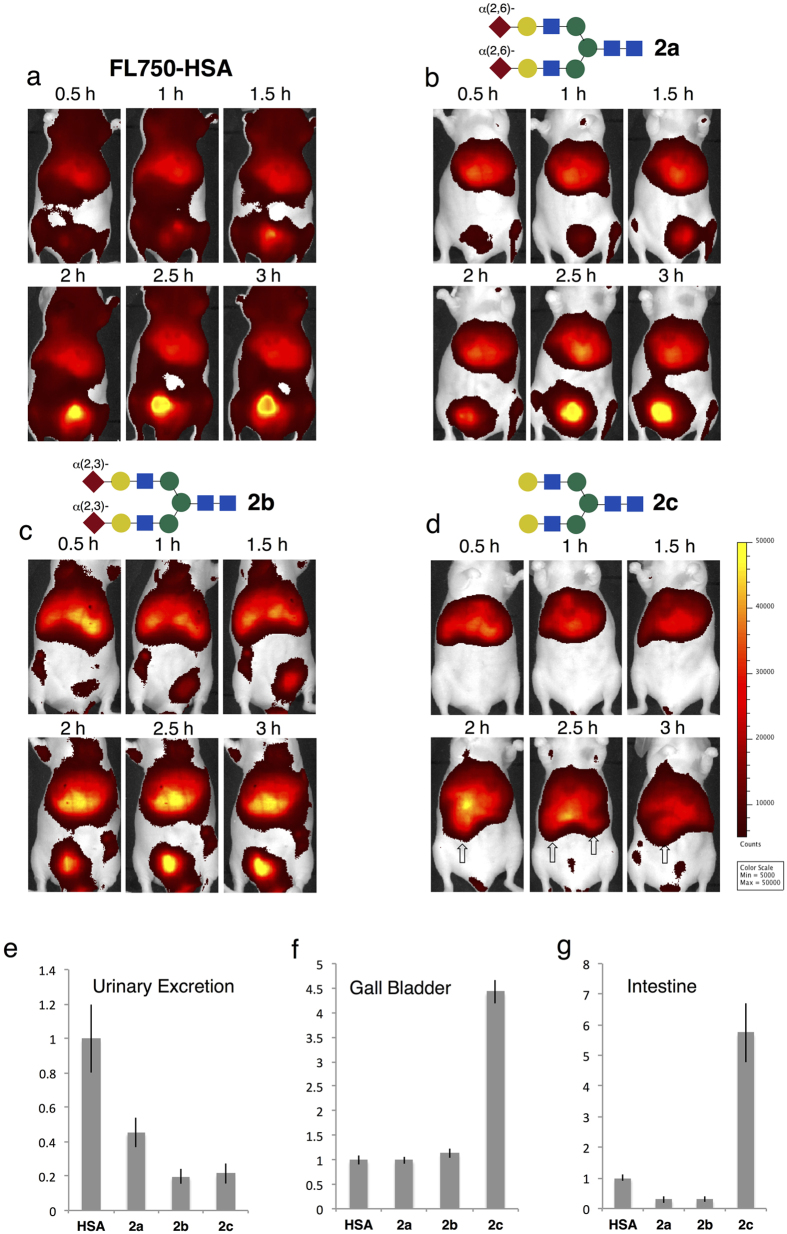
*In vivo* fluorescence imaging of *N*-glycoclusters 2a–c: Effects of terminal sialic acids and galactose on excretion rates and pathways. 1.5 nmol / 30 μL of *N*-glycoalbumins were diluted in 70 μL saline, and injected into 8 to 10-week-old BALB/cAJcl-nu/nu mice via the tail vein (N = 4). The mice were then anesthetized with pentobarbital or isoflurane and placed in a fluorescence imager. Abdominal images were taken at 30-minute intervals. After 3 hours of observation, the mice were sacrificed and perfused with 4% paraformaldehyde solution, and fluorescence intensities in the gall bladder and intestine were measured. (**a**–**d**): Fluorescence *in vivo* images (abdominal side) of (**a**) FL750-HSA, (**b**) α(2,6)-disialoglycoalbumin **2a**, (**c**) α(2,3)-disialoglycoalbumin **2b**, and (**d**) galactoglycoalbumin **2c**. Intestine is denoted by arrows. (**e**–**g**) Rate of fluorescent signal increased in (**e**) urinary bladder, fluorescence intensities of dissected (**f**) gall bladder and (**g**) intestine after 3 hours. Fluorescence was calculated within an arbitrarily defined region of interest (ROI). Mean values with standard errors, normalized to HSA, are indicated.

**Figure 4:**
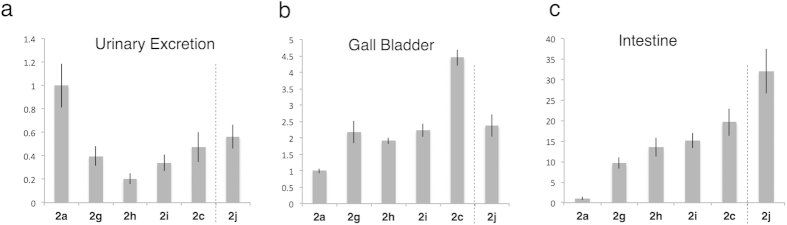
Excretion analysis of heterogeneous *N*-glycoclusters 2g–j: Excretion models of sialoglycoproteins by serum sialidase–mediated metabolism. Experiments were performed as described in Fig. 3 (see *in vivo* fluorescence images in Supplementary Information, Fig. S5). Rate of fluorescence signal accumulation in (**a**) urinary bladder, fluorescence intensities in dissected (**b**) gall bladder and (**c**) intestine after 3 hours. Fluorescence was calculated within an arbitrarily defined region of interest (ROI). Mean values with standard errors, normalized to **2a**, are indicated.

**Figure 5:**
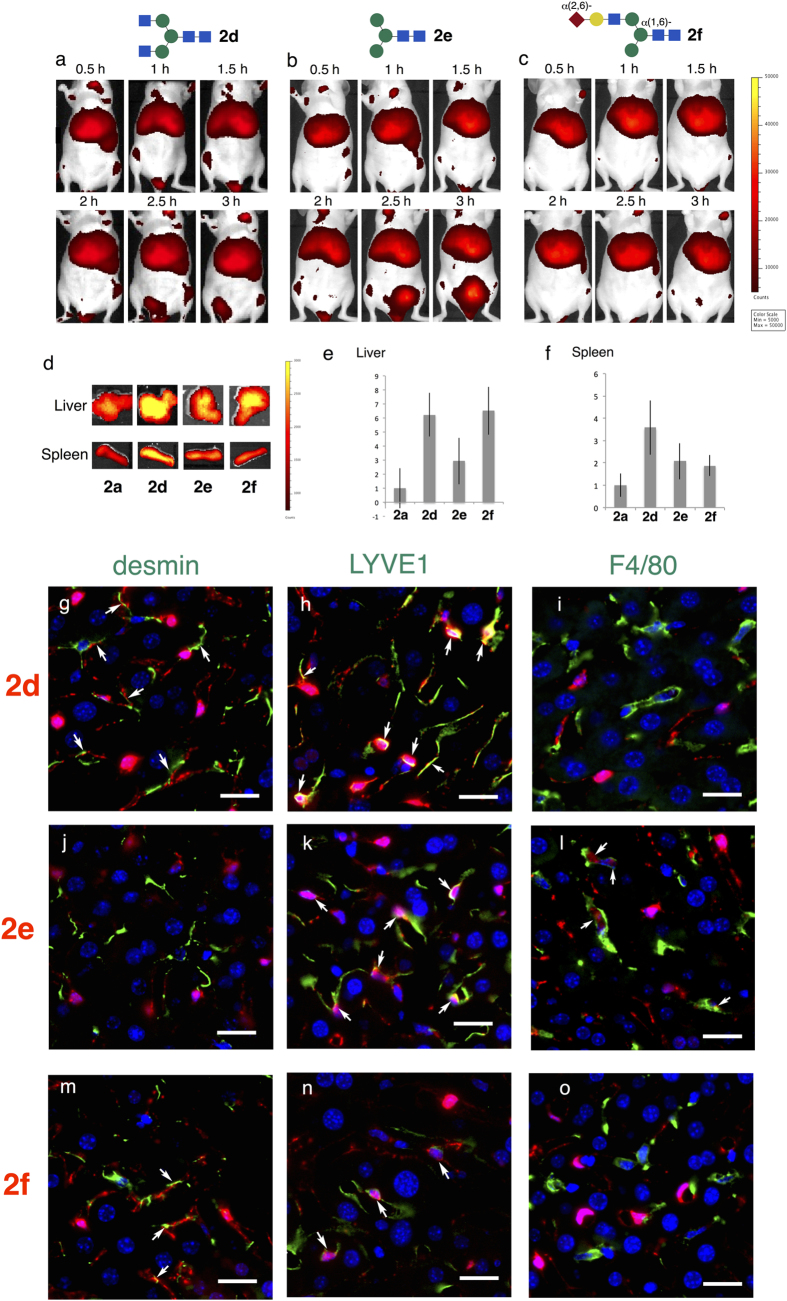
*In vivo* fluorescence imaging of *N*-glycoclusters 2d–f: Glycan-dependent accumulation to hepatic cells. (**a**–**c**): Fluorescence *in vivo* images (abdominal side) of (**a**) glucosamine **2d**, (**b**) mannose-terminated **2e**, and (**c**) hybrid-type glycoalbumin **2f**. (**d**–**f**): Fluorescence intensities accumulated at dissected (**e**) liver, within an arbitrarily defined region of interest (ROI), and (**f**) spleen after 3 hours. Mean values with standard errors, normalized to **HSA**, are indicated. (**g**–**o**): Co-staining of liver tissues treated with glucoalbumins **2d–f** with (**g**,**j**,**m**) anti-desmin (to detect stellate cells), (**h**,**k**,**n**) anti-LYVE1 (to detect sinusoidal endothelial cells), and (**i**,**l**,**o**) anti-F4/80 antibodies (to detect Kupffer cells and macrophages); Red: glycoalbumin; green: indicated antibodies; blue: nucleus. Scale bars represent 20 nm.

*N*-glycoalbumins prepared in this manner, labeled with near-infrared fluorescence (HiLyte Fluor 750^®^), were injected into BALB/cAJcl-nu/nu mice via the tail vein, and noninvasive fluorescence imaging was performed using IVIS^®^ under anesthesia. Although the fluorescence signals from the dorsal side did not exhibit any significant differences in trafficking and/or accumulation for any of the *N*-glycoalbumins investigated (i.e., the compounds appeared evenly distributed over the whole body; see Supplementary Information, Fig. S1), signals from the abdominal side clearly revealed time-dependent excretion as well as accumulation in specific organs. Consequently, *in vivo* kinetics and distribution are discussed using the abdominal data in Figs 3, 4, 5. After 3 h, the mice were dissected, and the exact localization of fluorescence signals was examined in representative organs. Some *N*-glycoalbumins exhibited strong accumulation in the liver (*vide infra*), and the collected livers were sliced and stained by the appropriate antibodies to identify the targets.

Thus, **FL750-HSA** without any *N*-glycans (as the control) was immediately distributed after the injection over the whole body through the capillary vessels on the skin, and then gradually excreted through the urinary bladder, typical of the *in vivo* kinetics of albumin (Fig. 3a)^30^. Albumin containing a few disialoglycans **a** behaved similarly to the native albumin, **FL750-HSA** (Supplementary Information, Fig. S2). The results are consistent with the observations of Gabius and co-workers, who showed that modification with a few *N*-glycans did not significantly alter the *in vivo* kinetics of albumin^17^.

By contrast, albumins covered with *N*-glycoclusters exhibited kinetics notably different from those of the control (**2a**–**j**, which contain about a dozen *N*-glycans); all of them initially trafficked to the liver within 30 min (Figs 3b–d and 5a–c), but after that, they exhibited either of two distinct properties: (i) specific excretion patterns (for glycoclusters **2a**–**c**) or (ii) target-selective accumulation (for glycoclusters **2d**–**f**). These properties appeared to depend critically on the *N*-glycan structures.

Regarding the former property, sialic acids and galactose at the non-reducing end of the glycan structures in **2a**–**c** significantly affected the rate and mechanism of excretion from the liver (Fig. 3). The rate of fluorescence signal accumulation in the urinary bladder, obtained by a semi-quantitative analysis of the excretion rate, is summarized in Fig. 3e. The α(2,6)- and α(2,3)-sialylated glycoalbumins **2a**,**b** were excreted through the bladder, and their fluorescence signals in the whole body gradually decreased over 24 h (data not shown), but at a much slower rate than the intact albumin **FL750-HSA**. These result are consistent with the well-known sialoside-dependent circulatory residence of glycoproteins^31^. Serum stabilization, especially by the α(2,3)-disialoside, has been reported for albumin modified with a few of *N*-glycan molecules^17^,^18^,^19^, but much more remarkable effects were observed due to the multivalency of our glycoalbumins.

On the other hand, instead of being cleared through the urinary bladder, the asialo-type glycocluster **2c**, a trimmed model of sialoglycan, was preferentially excreted *via* the gall bladder and the intestine (Fig. 3d,e). This was further confirmed by dissecting these organs 3 h after the injection and comparing the fluorescence intensities in the gall bladder and intestine with those of albumin or disialoglycans **2a**,**b** (Fig. 3f,g, also see Supplementary Information, Figs S3 and S4). Thus, the excretion pathways were completely altered by trimming of sialic acids on glycoalbumin.

We then examined the excretion properties of the heterogeneously glycosylated albumin **2g**–**j**, in which various ratios of α(2,6)-sialo- (**a**) and asialoglycans (**c**) were introduced (Fig. 2c); these heterogeneous clusters could thus mimic the partially de-sialylated soluble glycoproteins for asialoglycoprotein receptor (AGCR)-mediated excretion by the serum sialidases (Fig. 4). Although the effect was not uniform, in general we observed a shift of the excretion pathway away from urinary bladder (Fig. 4a) to the gall bladder/intestine (Fig. 4b,c), depending on the number of the sialic acids on glycoalbumins **2a**,**c**, and **g**–**i**. Specifically, the sequential trimming of non-reducing end sialic acids on proteins preferentially induced gall bladder excretion. It should be noted, however, that glycoalbumin **2j**, which was inversely clusterized by sialo- and asialoglycan-derived aldehydes (1 : 1 ratio of sialo- and asialoglycans, see details in Fig. 2b), exhibited much more rapid and complete intestinal excretion. Hence, not only the ratio of individual glycans clustered on glycoalbumin (obtained by simply summing each glycan function), but also the immobilized positions of each glycan, are very important for inducing the effects of heterogeneity.

Further trimmed glycoclusters, namely, glucosamine- (**2d**), mannose-terminated (**2e**), and hybrid (**2f**) glycoclusters, were not excreted via either the urinary bladder or the gall bladder. Instead, according to the second characteristic property defined above, these clusters exhibited organ-specific accumulation 0.5–3 h after injection (Fig. 5). Thus, the galactose-trimmed glycoalbumin **2d** (glucosamine-terminated) strongly accumulated in the liver and spleen (Fig. 5d–f), with fluorescence signals that were 6-fold higher than those of α(2,6)-disialoalbumin **2a** (used as a reference) in the dissected liver, and 4-fold higher in the spleen. The further trimmed mannose-terminated **2e** also accumulated in the liver and spleen, but to a lesser extent than the galactose-trimmed **2d** (3-fold higher accumulation in liver and 2-fold higher in spleen than the control, α(2,6)-disialoalbumin). Finally, the hybrid-type **2f**, which contains both α(2,6)-disialoside- and mannose-terminated branches on the glycan, did not exhibit the intermediate *in vivo* properties derived from these individual glycoalbumins **2a**,**e**, but instead strongly and selectively accumulated in the liver (6-fold higher than sialoglycoalbumin **2a**). In order to analyze the mechanisms underlying the high accumulation of the glucosamine, mannose and hybrid clusters (**2d**–**f**) in the liver, sliced sections were prepared from the livers collected by dissection after 3 hours, and the target cells were analyzed by immunostaining with specific antibodies (Supplementary Information). It should be noted that even the near-infrared dye, which can be detected with light at 780 nm, could be clearly detected using the newly developed Keyence All-in-one Fluorescence Microscope®. Thus, the trafficking and accumulation of fluorescence injected into the mice could directly be traced for the first time at the cellular level.

These glycoalbumins **2d**–**f** were not captured by parenchymal liver cells, i.e., through the asialoglycoprotein receptor (AGCR), but rather by non-parenchymal cells. Tissues stained with green-colored anti-desmin (for stellate cells)^32^ and anti-LYVE1 antibodies (for sinusoidal endothelial cells^33^, Fig. 5g,h) extensively co-localized with infrared fluorescence derived from glucosamine-terminated glycoalbumins **2d**, and to a lesser extent the anti-F4/80 antibody (for Kupffer cells^34^, Fig. 5i). Hybrid-type glycoalbumins **2f** exhibited similar properties (Fig. 5m–o). Therefore, liver stellate cells are largely responsible for the liver-specific accumulation of these glycoalbumins. Mannose-terminated glycoalbumin **2e**, on the other hand, did not overlap with anti-desmin; instead, due to the interaction with C-type lectins on sinusoidal endothelial and Kupffer cells^35^, it colocalized with LYVE1 and F4/80 (Fig. 5j–l), illustrating glycan-dependent accumulation in these cells.

## Discussion

Our imaging and tissue-based experiments clearly demonstrate the *N*-glycan–trimming dependence of *in vivo* kinetics and organ-specific accumulation of glycoalbumins, owing to glycan multivalency effects. As expected, the introduction of α(2,6)- and α(2,3)-disialoglycans increased the serum stability relative to that of intact albumin. Notably, asialoglycan shifted the excretion pathway from the urinary bladder to the gallbladder and intestine. Structurally well-defined heterogeneous glycoalbumins, containing various ratios of α(2,6)-sialo- and galactoglycans, clearly demonstrated this trend. Wong and Wu utilized microarrays to establish heterogeneous glycan environment to find out neighboring effect on antibody-glycan interaction^36^, but this is the first study to perform *in vivo* kinetics analysis of heterogeneous glycan clusters on protein surfaces. Our findings suggest that, in nature, sequential trimming of sialic acid from glycoproteins induces both urinary and intestinal excretion, which in turn might be accompanied by an increase of the total amount of protein excretion. Hence, even if not applicable or general to all natural glycoproteins, these phenomena could provide the first synthetic mimic of the clearance properties of *N*-glycoproteins: namely, asialoglycoproteins are readily excreted through the asialoglycoprotein receptor (AGCR) after trimming of sialic acid from sialoglycoproteins^4^,^5^.

Because both sialo- and asialoglycoalbumins were initially trafficked to the liver, there could be two distinct excretion mechanisms in liver cells. Asialoglycoalbumin could be recognized and endocytosed by AGCR in liver parenchymal cells, and then transported to the gallbladder via the polar transportation mechanism^37^. On the other hand, sialoglycoalbumin, due to the glycan multivalency effects on the albumin, could also weakly and reversibly bind to the AGCR^38^, and upon release, it could be metabolized for excretion into the urinary bladder through the biofiltration^39^. Future studies should attempt to determine the underlying mechanism.

GlcNAc-terminated, mannose-terminated, and hybrid-type glycoalbumins (**2d**–**f**) did not exhibit preferential excretion properties, but instead strongly accumulated in the liver. Surprisingly, our immunohistochemical study revealed that these glycoalbumins (**2d**–**f**) are not captured by parenchymal liver cells, but are instead captured preferentially by non-parenchymal cells. While mannose-terminated **2e** were captured by Kupper cells and/or macrophages through the interaction with C-type lectins^35^, in particular, glucosamine-terminated **2d** and hybrid-type **2f** mainly interacted with liver stellate cells as well as sinusoidal endothelial cells. This is entirely different from the behavior of, for instance, GlcNAc-based glycopolymers, which interact with AGCR in liver parenchymal cells, and monosaccharide-modified albumins, i.e., GlcNAc-albumin, which are simply washed out smoothly in comparison with other monosaccharide conjugates^40^. These results, in addition to the aforementioned excretion pathways that are regulated by Sia- and Gal-terminated *N*-glycoalbumins, highlight the value of a glycoalbumin strategy such as ours that utilizes whole *N*-glycan structures. In other words, the *in vivo* cluster effects of *N*-glycans cannot simply be mimicked by non-reducing end monosaccharides.

Very recently, Ise and co-workers found that their GlcNAc-terminated polymer materials can selectively interact with non-parenchymal cells, i.e., stellate, sinusoidal endothelial, and Kupffer cells^41^. Our GlcNAc-terminated glycoalbumin **2d** achieved these interactions at the whole-body level for the first time. Targeting of hepatic stellate cells is a highly desirable goal in liver treatment, because upon activation these cells play critical roles in liver fibrillation and cirrhosis^42^ or thrombotic thrombocytopenic purpura^43^. No promising tracers have been discovered to date. Ise found using their GlcNAc-terminated polymer materials that desmin and vimentin, usually recognized as cytoskeletal proteins, are expressed on the surface of liver stellate cells, and exhibit lectin activity especially on glucosamine^41^. Our results, which show that GlcNAc-terminated **2d**, as well as the unexpected discovered hybrid-type **2f**, can thus recognize the hepatic stellate cells *in vivo*, suggest that these *N*-glycoalbumins represent a breakthrough for diagnosis and treatment of liver fibrillation.

Thus, *in vivo* kinetics and organ-specific accumulation of our glycoclusters are not consistent with those reported by Gabius and co-workers^17^,^18^, where a few *N*-glycans were introduced onto albumin. In fact, natural glycoproteins generally have a few *N*-glycans, hence our glycoclusters immobilized with as many as a dozen complex *N*-glycans might exaggerate or even alter real recognition processes of natural glycoproteins. Nevertheless, these new finding of notable *N*-glycan–trimming dependence of our glycoalbumins will provide useful information in elucidating and applying the extracellular *N*-glycan functions, in terms of exploring the complex protein- and cell-surface pattern recognition *in vivo*.

## Conclusion

We developed a highly effective one-pot glycan conjugation strategy for proteins. With this conjugation methodology, we successfully analyzed the dependence of the kinetics of various *N*-glycoalbumin on non-reducing glycans. Our results were obtained by noninvasive fluorescence imaging and microscopic analyses of the dissected tissues. Extracellular trimming of *N*-glycans trigger pathway-selective excretion or cell-specific targeting, revealing previously unappreciated functions of ubiquitous *N*-glycans. These results should be broadly applicable in extracellular analysis of *N*-glycan functions, and raise the possibility of diagnostic and therapeutic applications.

## Methods

To a 9 mM solution of **a** (glycan-azide) (1.8 × 10^−7 ^mol, 36 eq) in DMSO was added 5 mM MeCN solution of **1** (1.5 × 10^−7 ^mol, 30 eq) under a nitrogen atmosphere. The reaction mixture was heated to 70 °C and monitored by HPLC. After consumption of the starting aldehyde, the reaction mixture was cooled down to RT, and diluted with DMSO and water. Subsequently, **HLF-HSA** stock solution (5.0 × 10^−9 ^mol, 1 eq) was added and mixed well. The reaction mixture was incubated at 37 °C overnight with continuous mild shaking. The resultant mixture was centrifuged with Amicon® 10 K and subsequently washed three times with water. The solution above the membrane was filtered with Durapore® PVDF 0.45 μm and diluted with pure water up to a final volume of 100 μl to give solution **2a** (50 mM). For animal experiments, 30 μl of solution **2a** was diluted with 70 μl saline. Complete experimental procedures and characterization are described in the Supplementary Information.

All procedures involving experiment animals were approved by the Ethics Committee of RIKEN (MAH21-19-17). The experiments were performed in accordance with the institutional and national guidelines.

## Additional Information

**How to cite this article**: Ogura, A. *et al.* Visualizing Trimming Dependence of Biodistribution and Kinetics with Homo- and Heterogeneous *N*-Glycoclusters on Fluorescent Albumin. *Sci. Rep.* **6**, 21797; doi: 10.1038/srep21797 (2016).

## Supplementary Material

Supplementary Information

## Figures and Tables

**Figure 1:**
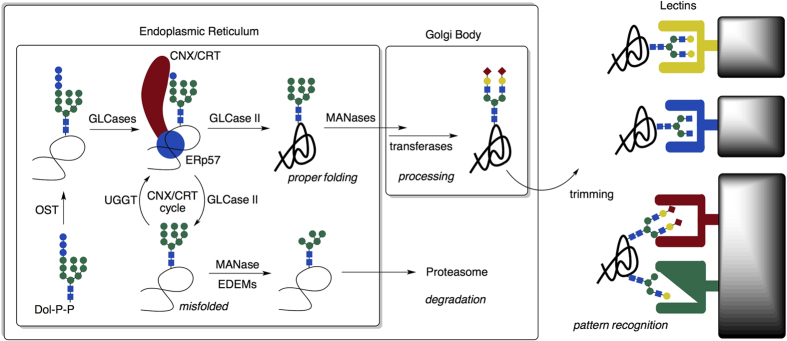
Intracellular and extracellular functions of *N*-glycans. Even though the intracellular role of *N*-glycans in protein quality control has been well studied (left), the extracellular functions of *N*-glycans are not clear. Partial trimming confers glycan structural diversity on proteins or cells, including heterogeneous glycoclusters, which are selectively recognized by specific extracellular lectins (right).
