# Supplementary material for: Visualizing Trimming Dependence of Biodistribution and Kinetics with Homo- and Heterogeneous N-Glycoclusters on Fluorescent Albumin
Source: Sci Rep. 2016 Feb 23;6:21797. doi: 10.1038/srep21797 (PMC4763176; doi:10.1038/srep21797)
Supplement: Supplementary Information [file srep21797-s1.pdf]

Supplementary Information

Visualizing Trimming Dependence of Biodistribution and Kinetics with Homo- and Heterogeneous *N*-Glycoclusters on Fluorescent Albumin

Akihiro Ogura[1], Tsuyoshi Tahara[2], Satoshi Nozaki[2], Koji Morimoto[3], Yasuhiko Kizuka[4], Shinobu Kitazume[4], Mitsuko Hara[5], Soichi Kojima[5], Hirotaka Onoe[2], Almira Kurbangalieva[6], Naoyuki Taniguchi[4], Yasuyoshi Watanabe[2] & Katsunori Tanaka*[1,6,7]

1. Biofunctional Synthetic Chemistry Laboratory, RIKEN, 2-1 Hirosawa, Wako-shi, Saitama 351-0198, Japan

2. RIKEN Center for Life Science Technologies, 6-7-3 Minatojima-minamimachi, Chuo-ku, Kobe, Hyogo 650-0047, Japan

3. Osaka Women's Junior College, 3-8-1 Kasugaoka, Fujiidera-shi, Osaka, 583-8558, Japan

4. Disease Glycomics Team, Global Research Cluster, RIKEN-Max Planck Joint Research Center for Systems Chemical Biology, RIKEN, 2-1 Hirosawa, Wako-shi, Saitama 351-0198, Japan

5. Micro-Signaling Regulation Technology Unit, RIKEN Center for Life Science Technologies, Wako-shi, Saitama, 351-0198, Japan

6. Biofunctional Chemistry Laboratory, A. Butlerov Institute of Chemistry, Kazan Federal University, 18 Kremlyovskaya Street, Kazan 420008, Russia

7. Japan Science and Technology Agency-PRESTO, 2-1 Hirosawa, Wako-shi, Saitama 351-0198, Japan

Corresponding author: Katsunori Tanaka (e-mail: kotzenori@riken.jp)

1. Chemical Synthesis

1.1. General 3

1.2. Preparation of glycoalbumins 4

2. *In Vivo* Kinetics and Biodistribution Analysis

2.1. General 30

2.2. Supplementary images 31

3. Immunohistochemistry 36

4. References 37

1. Chemical Synthesis

1.1. General

All commercially available reagents were used without further purification. Aldehyde **1** was prepared as previously described.[1] All *N*-glycans were provided by Glytech, Inc. Azide-functionalized *N*-glycans **a**-**f** were prepared according to the previously reported procedure.[2] Ultrapure water from Merck Milli-Q Advantage® was used for all synthetic experiments described in this paper. Reverse phase HPLC analysis/purification was performed on a Shimadzu Prominence® system equipped with a Nacalai tesque column (5C18-AR-300, 4.6 x 250 mm). Two solvent systems, namely, A: H_2O containing 0.1% TFA and B: MeCN containing 0.1% TFA, were applied. High-resolution mass spectra (HRMS) were obtained on a Bruker micrOTOF-QIII spectrometer® by electron spray ionization (ESI-TOF-MS). Mass spectra of the glycan-conjugated albumins were obtained on a Bruker autoflex spectrometer® by matrix assisted laser desorption ionization (MALDI-TOF MS), using 2,5-dihydroxybenzoic acid as a matrix.

1.2. Preparation of glycoalbumins

Preparation of α(2,6)-disialoglycan-modifying probe **1a** (prepared as the stock solution to be used below for homogeneous and heterogeneous glycoalbumin synthesis)

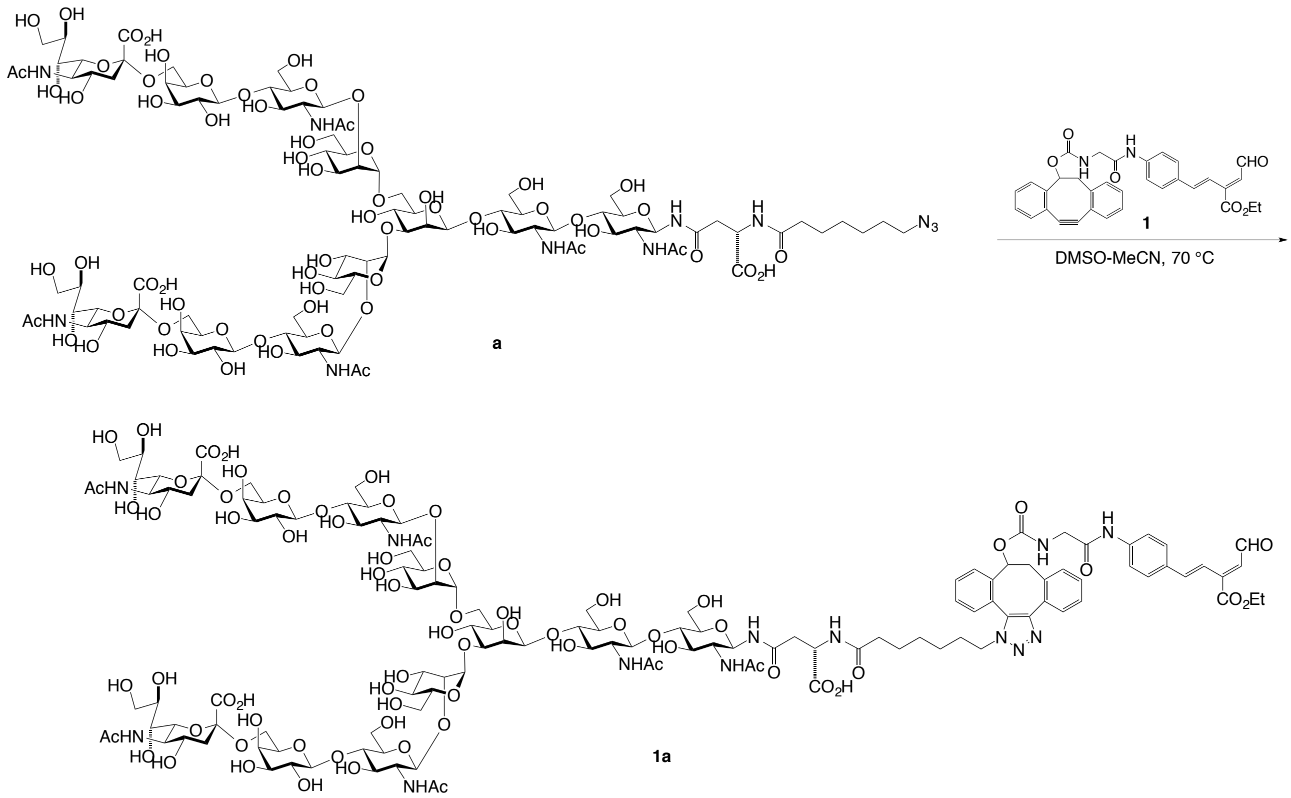

To a solution of α(2,6)-disialoglycan **a** (1.2 mg, 0.50 μmol) in DMSO (139 μL) was added 10 mM solution of aldehyde **1** in MeCN (45 μL, 0.45 μmol) under nitrogen atmosphere. The resulting mixture was heated to 70 °C and monitored by HPLC (gradient: from 10% B/90% A to 100% B over 30 min. While the aldehyde **1** was detected at 27.5 min, the clicked product **1a** was observed at 17.7 and 17.9 min (regioisomers at triazole ring)). After the consumption of the starting aldehyde **1**, the mixture was cooled down to RT to give 3.8 mM stock solution of **1a**, which was analyzed by ESI-HRMS (detected: 1518.0509, calcd: 1518.0482 for $C_{128}H_{183}N_{13}O_{71}$ $[M-2H]^{2-}$).

Preparation of α(2,3)-disialoglycan-modifying probe **1b** (prepared as the stock solution to be used below for homogeneous glycoalbumin synthesis)

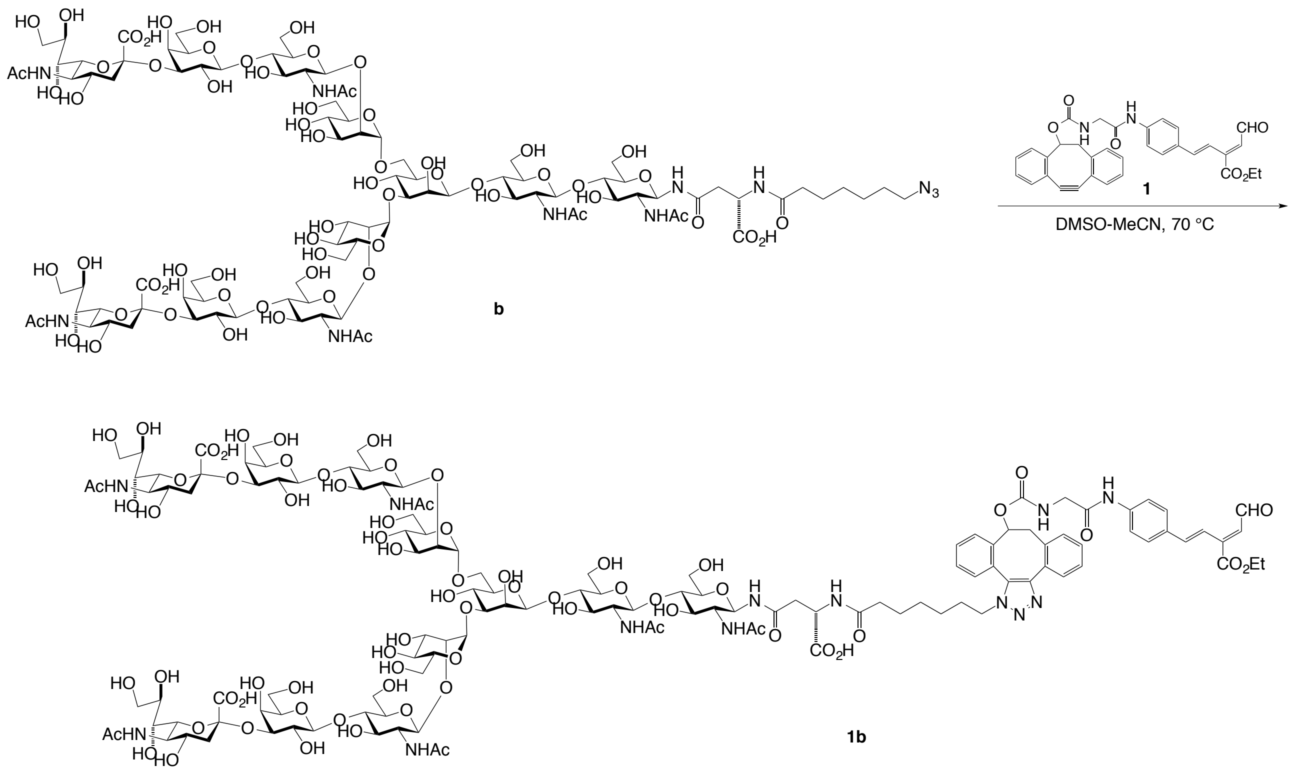

To a solution of α(2,3)-disialylglycan **b** (1.5 mg, 0.59 μmol) in DMSO (144 μL) was added 10 mM solution of aldehyde **1** in MeCN (54 μL, 0.54 μmol) under nitrogen atmosphere. The reaction mixture was heated to 70 °C and monitored by HPLC (gradient: from 10% B/90% A to 100% B over 30 min. The product **1b** was detected at 17.7 and 17.9 min). After the consumption of the starting aldehyde **1**, the mixture was cooled down to RT to give 3.8 mM stock solution of **1b**, which was analyzed by ESI-HRMS (detected: 1518.0460, calcd: 1518.0482 for $C_{128}H_{183}N_{13}O_{71}$ $[M-2H]^{2-}$).

Preparation of asialoglycan-modifying probe **1c** (prepared as the stock solution to be used below for homogeneous and heterogeneous glycoalbumin synthesis)

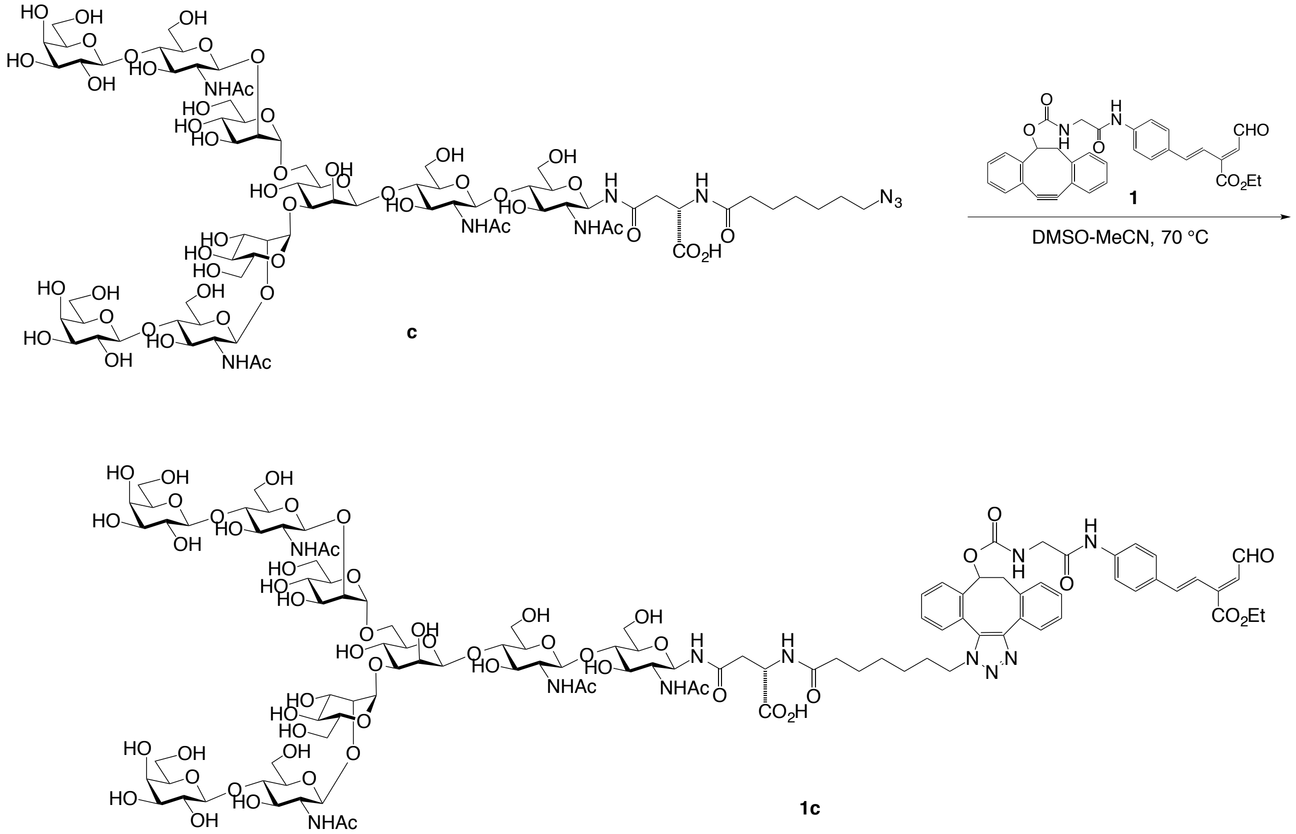

To a solution of asialoglycan **c** (1.1 mg, 0.57 μmol) in DMSO (139 μL) was added 10 mM solution of the aldehyde **1** in MeCN (52 μL, 0.52 μmol) under nitrogen atmosphere. The resulting mixture was heated to 70 °C and monitored by HPLC (gradient: from 10% B/90% A to 100% B over 30 min. The clicked product **1c** was detected at 18.0 and 18.2 min). After disappearance of the starting aldehyde **1**, the reaction mixture was cooled down to RT to give 3.8 mM stock solution of **1c**, which was analyzed by ESI-HRMS (detected: 1226.9545, calcd: 1226.9527 for $C_{106}H_{147}N_{11}O_{55}$ $[M-2H]^{2-}$).

Preparation of stock solution of **FL750-HSA**

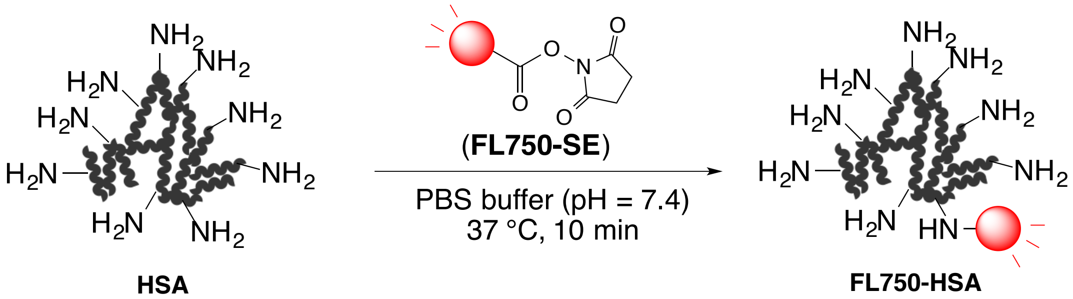

To a solution of human serum albumin (HSA, purchased from SIGMA, 3.2 mg, 48 nmol) in phosphate saline buffer (pH = 7.4, 300 μL) was added HiLyte Fluor 750 acid SE® (0.25 mg, 0.19 μmol, tetraethylammonium salt) in DMSO (10 μL) and the mixture was warmed to 37 °C. After 10 min, the solution was centrifuged through Amicon 10K® at 15,000 rpm for 10 min to filter off the small molecules. The residue was further washed with phosphate buffer and centrifuged for three times. The resulting solution was diluted by ultrapure water to 800 μL to afford the stock solution of **FL750-HSA**, which was used for subsequent *N*-glycan modification. MALDI-TOF-MS (positive mode) detected the molecular weight of **FL750-HAS** at 70.5 kDa, which contains average number of HiLyte Fluor 750, 3.1 fluorophores per albumin molecule.

Preparation of α(2,6)-disialylated glycoalbumin **2a**

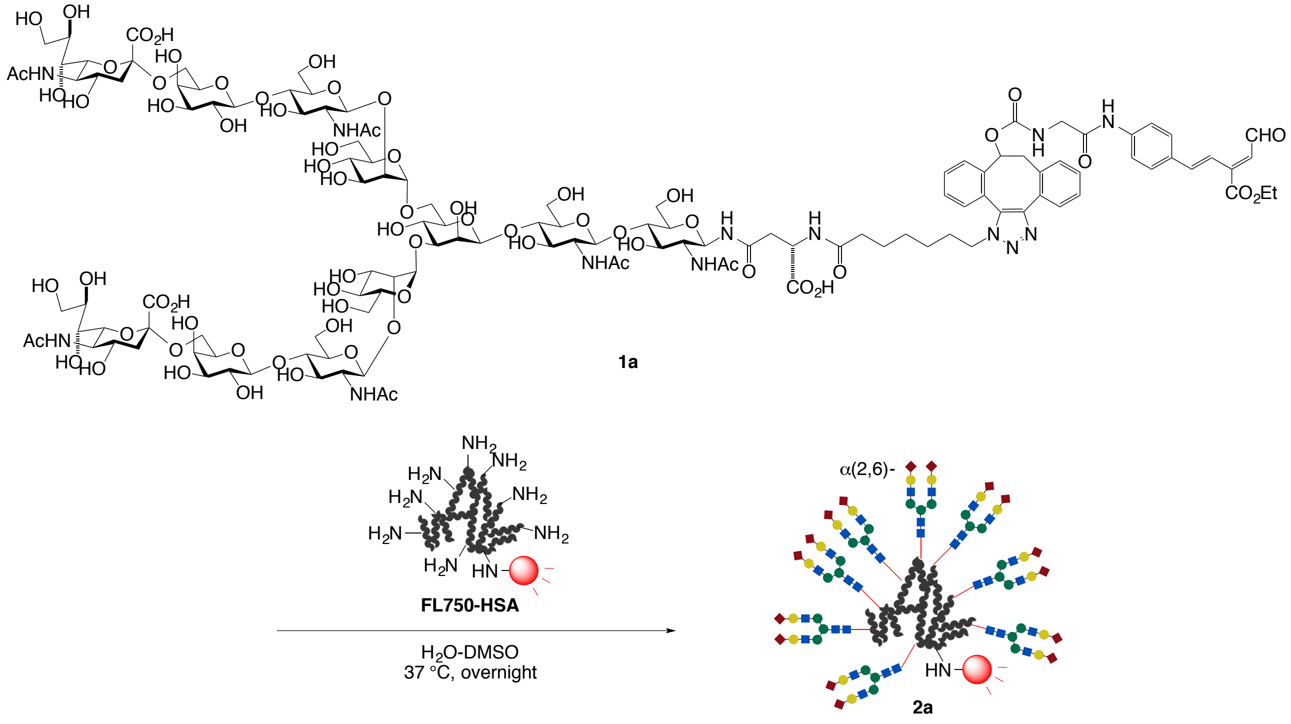

To **FL750-HSA** stock solution obtained above (132 µL, 7.5 nmol) was added water (132 µL), DMSO (66 µL), and then 3.8 mM stock solution of **1a** prepared above (0.12 µmol, 16 eq) in DMSO (32 µL) in two portions under air. The mixture was incubated overnight at 37 °C. The resulting solution was centrifuged through Amicon 10K® at 15,000 rpm for 10 min, and further washed with water three times to filter off any small molecules. The insoluble byproducts were further removed by filtering with Durapore PVDF 0.45 µm® and diluted with water to give 150 µL solution of α(2,6)-disialylated glycoalbumin **2a**. MALDI-TOF-MS (positive mode) detected the molecular weight of **2a** at 98.0 kDa, which contains average number, 9.2 molecules of glycan **a** per albumin.

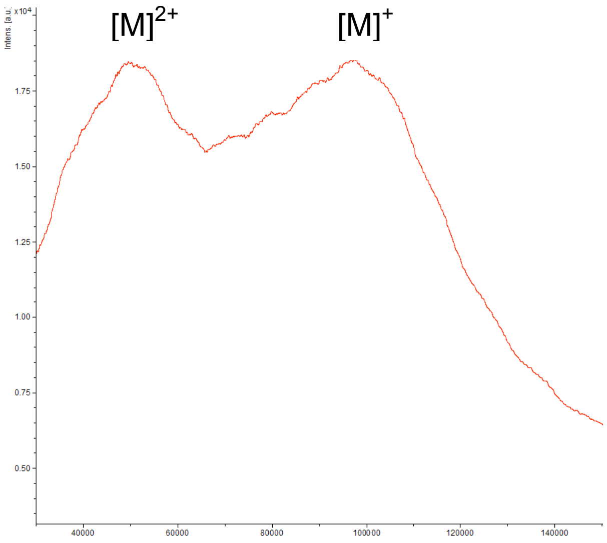

MALDI-TOF-MS of 2a

Preparation of α(2,3)-disialylated glycoalbumin **2b**

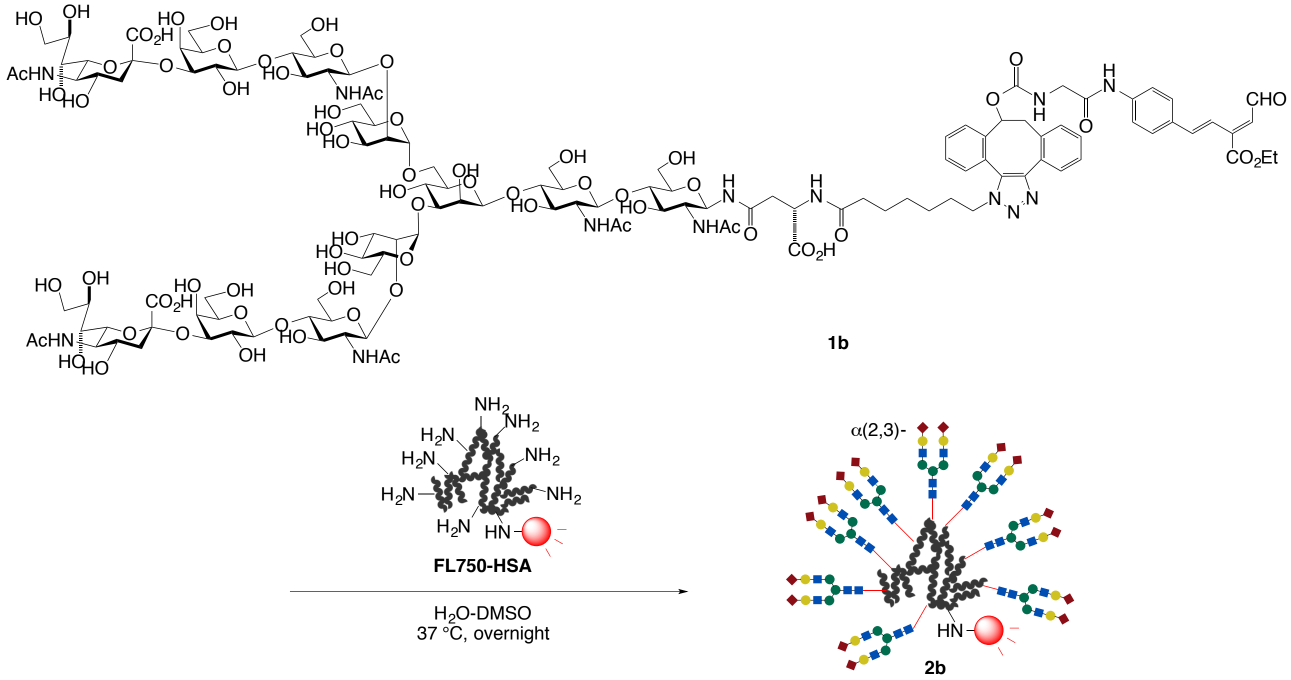

To **FL750-HSA** stock solution obtained above (52.5 μL, 3.0 nmol) was added water (52.5 μL), DMSO (26.2 μL), and then 3.8 mM stock solution of **1b** prepared above (90 nmol, 30 eq) in DMSO (24 μL) under air. The reaction mixture was incubated overnight at 37 °C. The resulting solution was centrifuged through Amicon 10K® at 15,000 rpm for 10 min, and further washed with water three times to filter off any small molecules. The insoluble byproducts were further removed by filtering with Durapore PVDF 0.45 μm® and diluted with water to give 60 μL solution of α(2,3)-disialylated glycoalbumin **2b**. MALDI-TOF-MS (positive mode) detected the molecular weight of **2b** at 102.1 kDa, which contains average number, 10.5 molecules of glycan **b** per albumin.

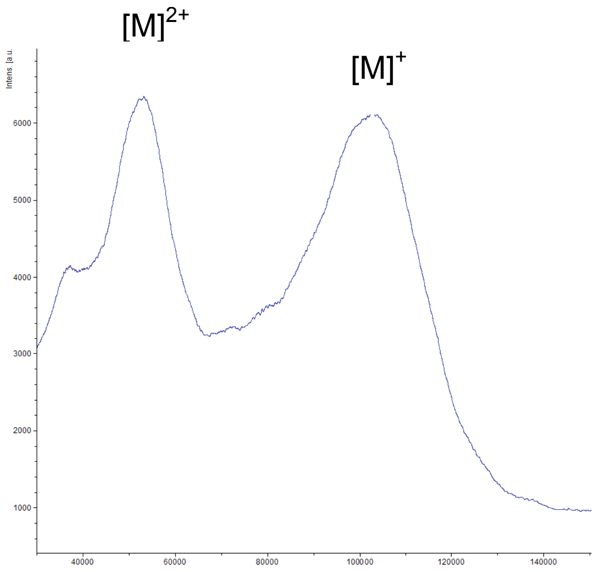

MALDI-TOF-MS of 2b

Preparation of asialoglycoalbumin **2c**

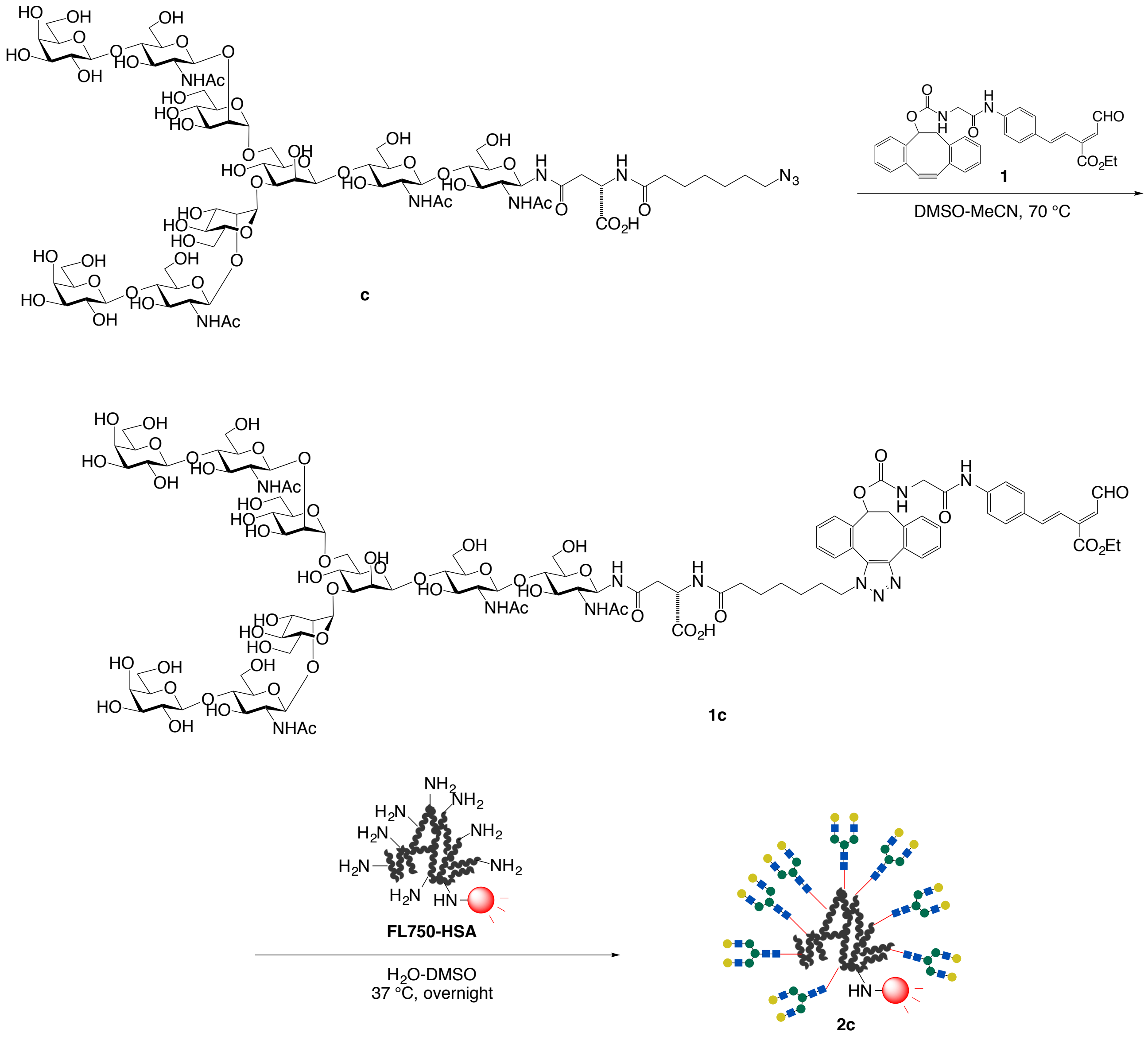

NOTE: one-pot glycoalbumin synthesis is described here. Following the procedure for preparation of stock solution **1c** described above, to a solution of asialoglucan **c** (0.29 mg, 0.15 μmol) in DMSO (20 μL) was added 5 mM solution of the aldehyde **1** in MeCN (30 μL, 0.15 μmol) under nitrogen atmosphere. The mixture was heated to 70 °C and monitored by HPLC After the consumption of the starting aldehyde **1**, the mixture was cooled down to RT, and diluted with DMSO (44 μL) and water (88 μL). Subsequently, **FL750-HSA** stock solution obtained above (88 μL, 5.0 nmol) was added and the resulting mixture was incubated overnight at 37 °C. The resulting solution was centrifuged through Amicon 10K®

at 15,000 rpm for 10 min, and further washed with water three times to filter off any small molecules. The insoluble byproducts were further removed by filtering with Durapore PVDF 0.45 μm® and diluted with water to give 100 μL solution of asialoglycoalbumin **2c**. MALDI-TOF-MS (positive mode) detected the molecular weight of **2c** at 92.6 kDa, which contains average number, 9.1 molecules of glycan **c** per albumin.

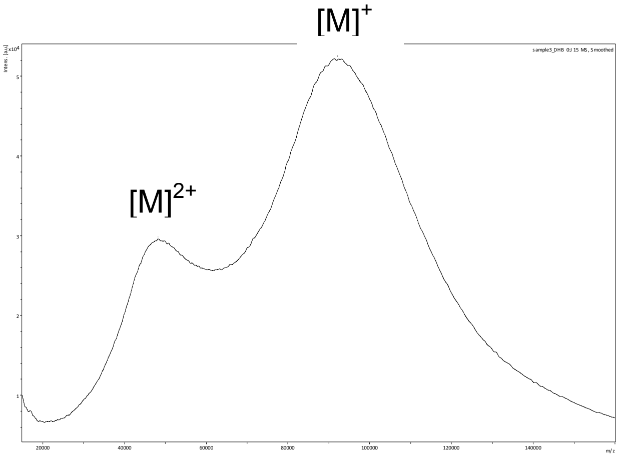

MALDI-TOF-MS of 2c

Preparation of glucosamine-terminated glycoalbumin **2d**

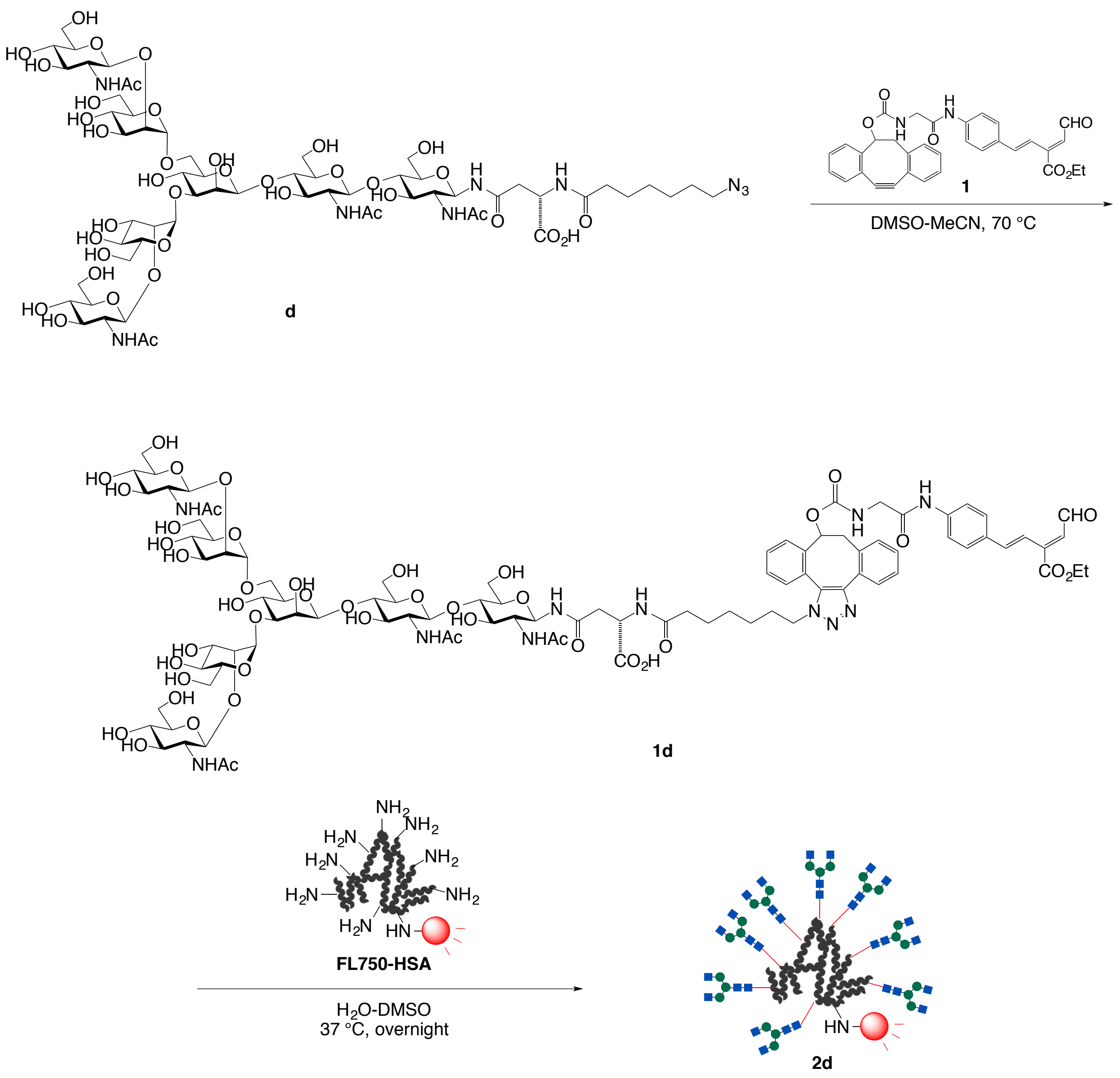

To a solution of *N*Ac-glucosamine-terminated glycan **d** (0.24 mg, 0.15 μmol) in DMSO (20 μL) was added 5 mM solution of aldehyde **1** in MeCN (30 μL, 0.15 μmol) under nitrogen atmosphere. The reaction mixture was heated to 70 °C and monitored by HPLC (gradient: from 10% B/90% A to 100% B over 30 min). The clicked product **1d** was detected at 18.3 and 18.5 min (ESI-HRMS; detected: 1064.9041, calcd: 1064.8999 for $C_{94}H_{129}N_{11}O_{45}$ $[M-2H]^{2-}$). After the consumption of the starting aldehyde, the mixture was cooled down to RT, and diluted with DMSO (44 μL) and water (88 μL). Subsequently, **FL750-HSA** stock

solution prepared above (88 μL, 5.0 nmol) was added and the resulting mixture was incubated overnight at 37 °C. The resulting solution was centrifuged through Amicon 10K® at 15,000 rpm for 10 min, and further washed with water three times to filter off any small molecules. The insoluble byproducts were further removed by filtering with Durapore PVDF 0.45 μm® and diluted with water to give 100 μL solution of glucosamine-terminated glycoalbumin **2d**. MALDI-TOF-MS (positive mode) detected the molecular weight of **2d** at 91.9 kDa, which contains average number, 10.1 molecules of glycan **d** per albumin.

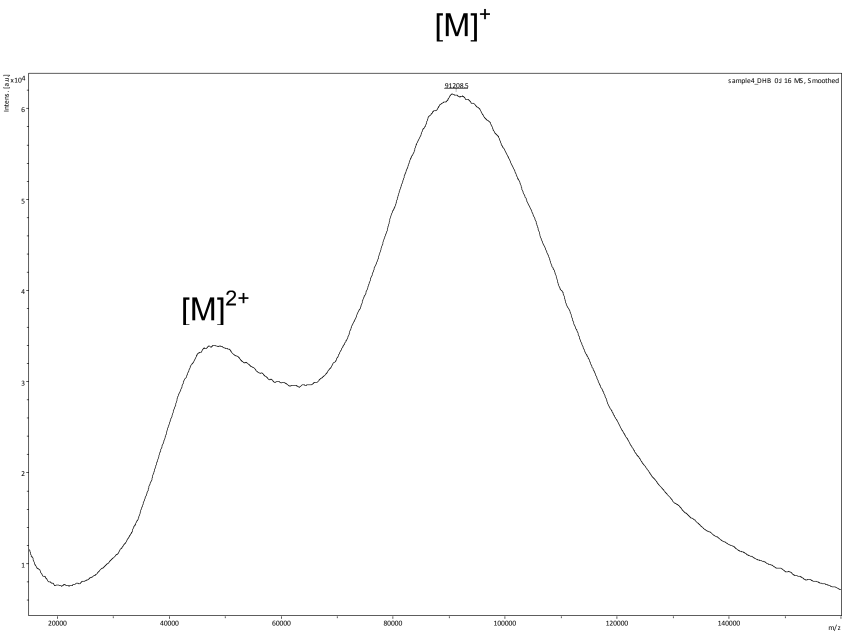

MALDI-TOF-MS of 2d

Preparation of mannose-terminated glycoalbumin **2e**

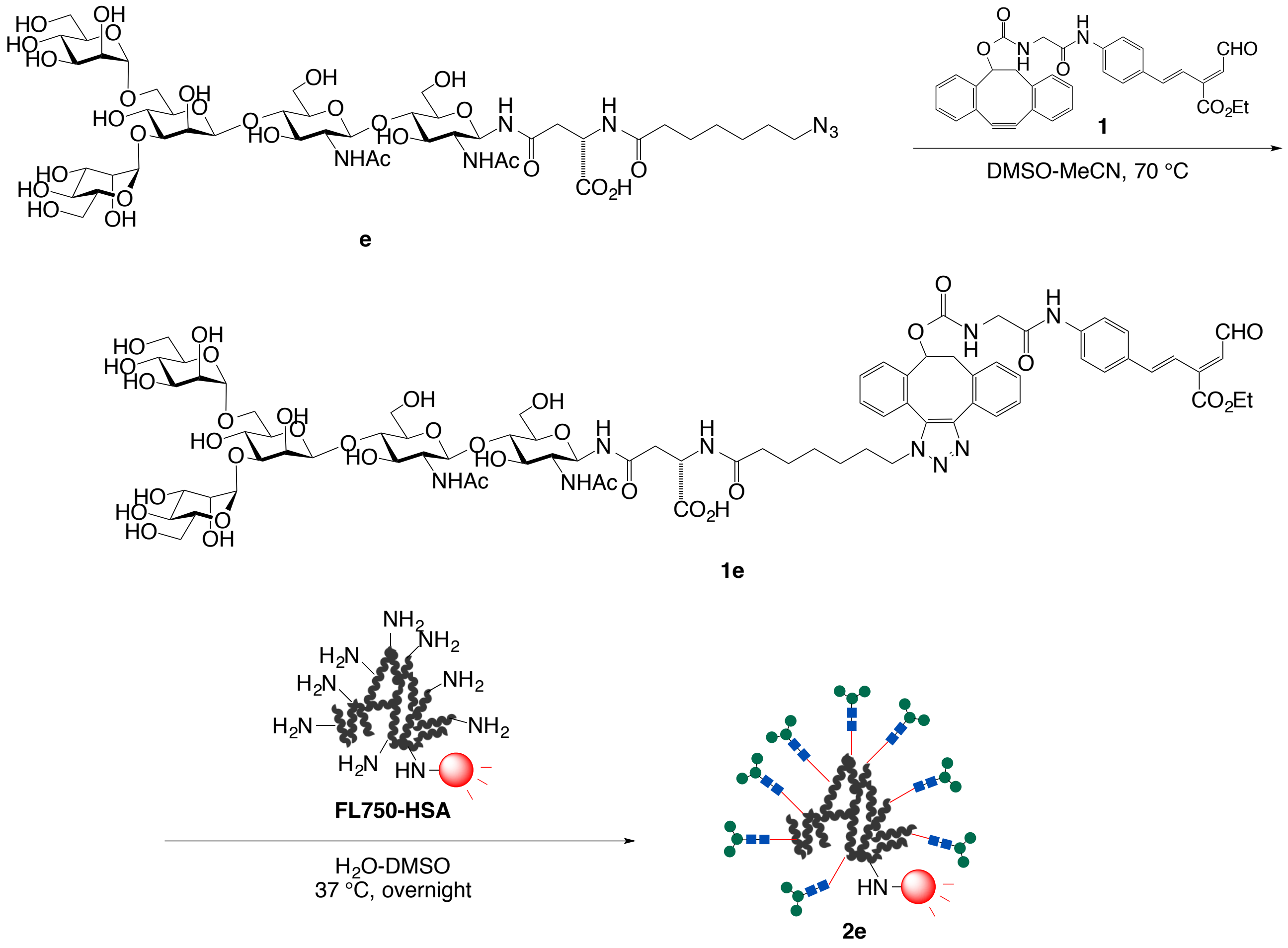

To a solution of mannosamine-terminated glycan **e** (0.18 mg, 0.15 μmol) in DMSO (20 μL) was added 5 mM solution of the aldehyde **1** in MeCN (30 μL, 0.15 μmol) under nitrogen atmosphere. The reaction mixture was heated to 70 °C and monitored by HPLC (gradient: from 10% B/90% A to 100% B over 30 min). The clicked product **1e** was detected at 18.5 and 18.7 min (ESI-HRMS; detected: 861.8176, calcd: 861.8206 for $C_{78}H_{101}N_9O_{35}$ $[M-2H]^{2-}$). After the disappearance of the starting aldehyde **1**, the mixture was cooled down to RT, and diluted with DMSO (44 μL) and water (88 μL). Subsequently, **FL750-HSA** stock solution prepared above (88 μL, 5.0 nmol) was added and the resulting solution was incubated overnight at 37 °C. The mixture was centrifuged through Amicon 10K® at 15,000 rpm for 10 min, and further washed with water three times to filter off any small molecules. The insoluble byproducts were further removed by filtering with Durapore

PVDF 0.45 μm® and diluted with water to give 100 μL solution of mannose-terminated glycoalbumin **2e**. MALDI-TOF-MS (positive mode) detected the molecular weight of **2e** at 88.5 kDa, which contains average number, 10.4 molecules of glycan **e** per albumin.

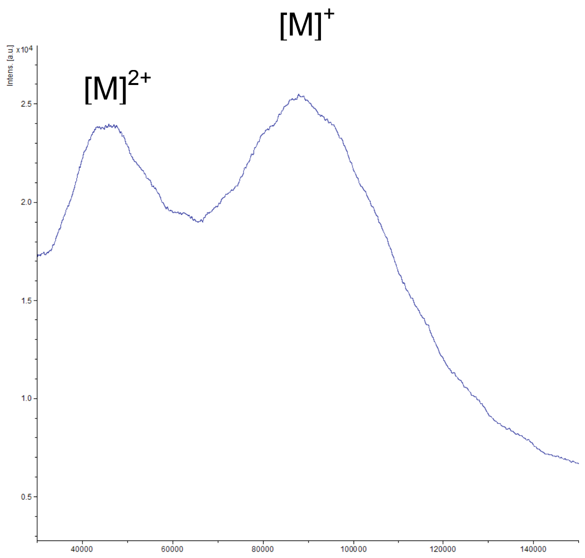

MALDI-TOF-MS of 2e

Preparation of hybrid-type glycoalbumin **2f**

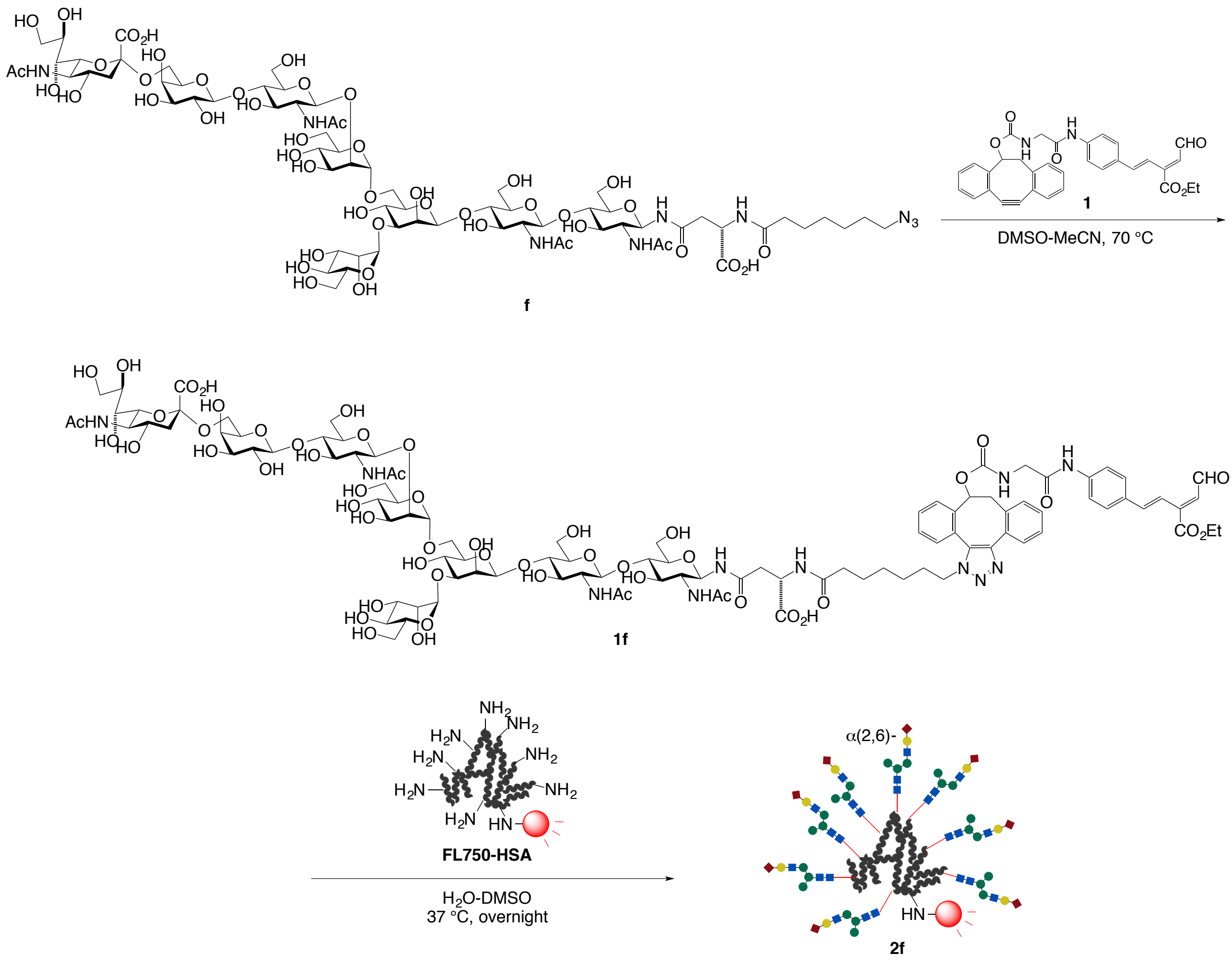

To a solution of hybrid-type glycan **f** (0.28 mg, 0.15 μmol) in DMSO (20 μL) was added 5 mM solution of the aldehyde **1** in MeCN (30 μL, 0.15 μmol) under nitrogen atmosphere. The reaction mixture was heated to 70 °C and monitored by HPLC (gradient: from 10% B/90% A to 100%B over 30 min). The corresponding clicked product **1f** was detected at 18.0 and 18.2 min (ESI-HRMS; detected: 1189.9316, calcd: 1189.9344 for $C_{103}H_{143}N_{11}O_{53}$ $[M-2H]^{2-}$). After the consumption of the starting aldehyde **1**, the mixture was cooled down to RT, and diluted with DMSO (44 μL) and water (88 μL). Subsequently, **FL750-HSA** stock solution obtained above (88 μL, 5.0 nmol) was added and the mixture was incubated overnight at 37 °C. The resulting solution was centrifuged through Amicon 10K® at 15,000

rpm for 10 min, and further washed with water three times to filter off any small molecules. The insoluble byproducts were further removed by filtering with Durapore PVDF 0.45 μm® and diluted with water to give 100 μL solution of hybrid-type glycoalbumin **2f**. MALDI-TOF-MS (positive mode) detected the molecular weight of **2f** at 94.0 kDa, which contains average number, 9.9 molecules of glycan **f** per albumin.

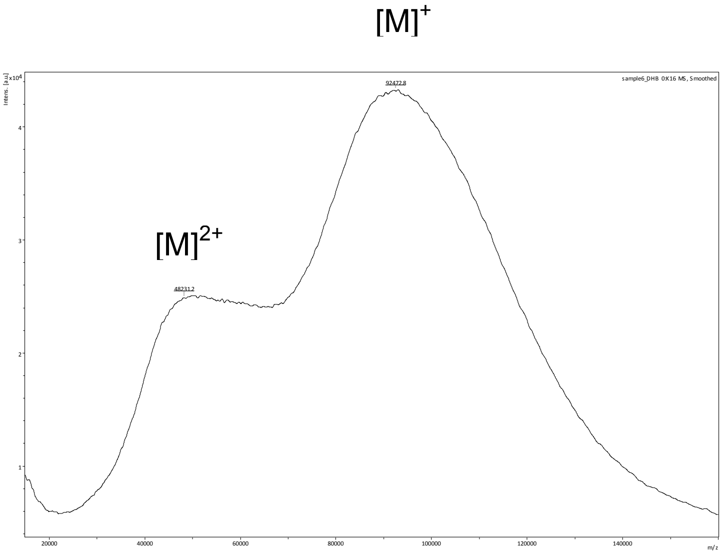

MALDI-TOF-MS of 2f

Preparation of α(2,6)-disialylated glycoalbumin **2a’** with fewer glycan

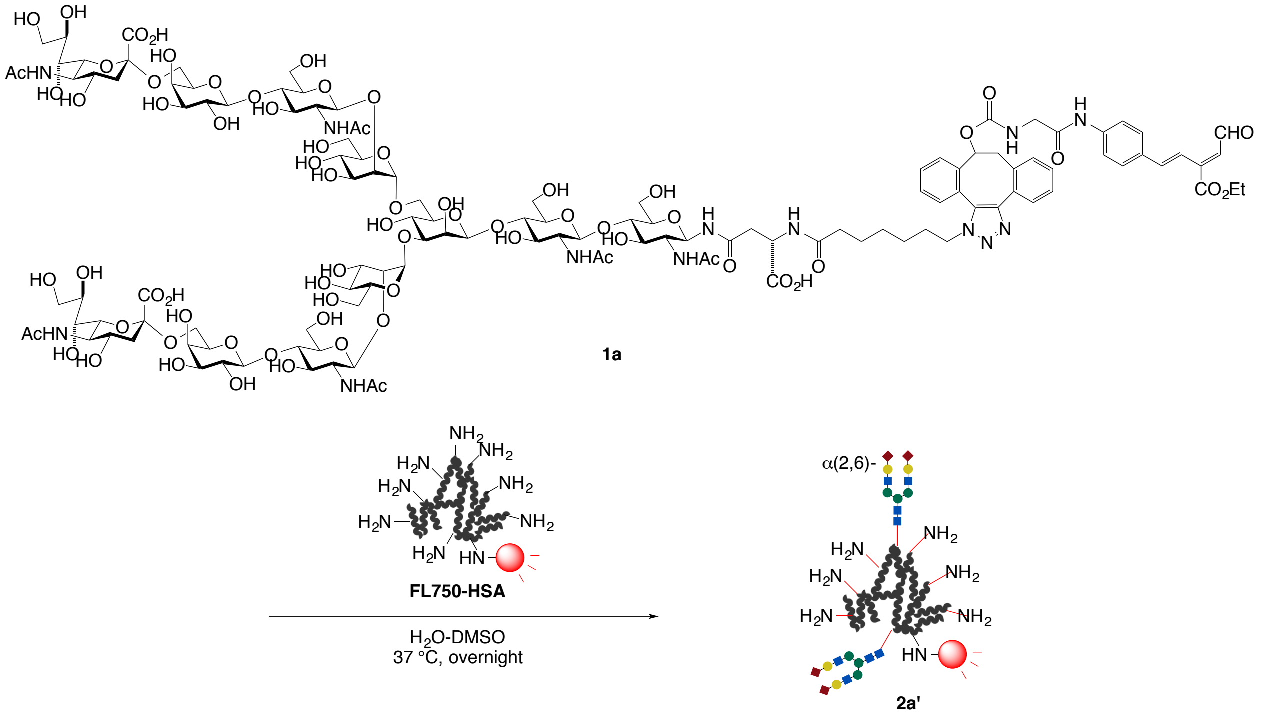

To a solution of α(2,6)-disialoglycan **a** (0.10 mg, 40 nmol) in DMSO (20 μL) was added 10 mM MeCN solution of the aldehyde **1** (2.5 μL, 25 nmol) under nitrogen atmosphere. The reaction mixture was heated to 70 °C and the completion of the reaction was evaluated by HPLC (see above). The clicked solution containing **1a** was then cooled down to RT, and diluted with DMSO (44 μL) and water (88 μL). Subsequently, **FL750-HSA** solution (88 μL, 5.0 nmol) was added and the mixture was incubated overnight at 37 °C. The resulting solution was centrifuged through Amicon 10K® at 15,000 rpm for 10 min, and further washed with water three times to filter off any small molecules. The insoluble byproducts were then removed by filtering with Durapore PVDF 0.45 μm® and diluted with water to give 100 μL solution of α(2,6)-disialylated glycoalbumin **2a’** (with a few glycans immobilized). MALDI-TOF-MS (positive mode) detected the molecular weight of **2a’** at 76.0 kDa, which contains average number, 1.8 molecules of glycan **a** per albumin.

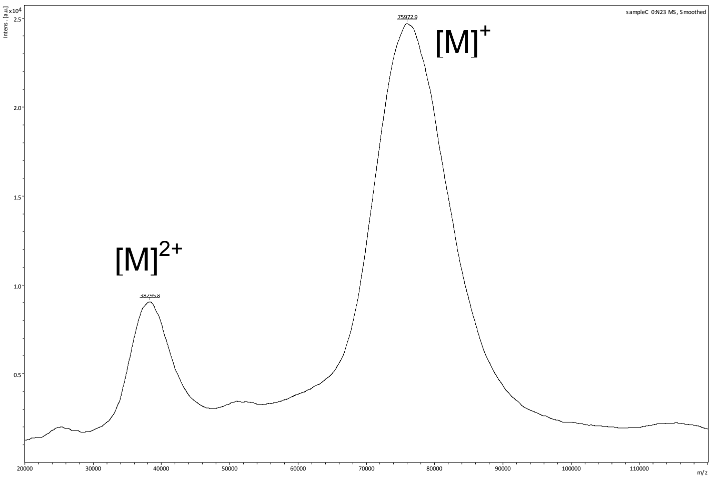

MALDI-TOF-MS of 2a’

Preparation of heterogeneous glycoalbumin **2g** with α(2,6)-disialo- and asialoglycans (8 : 2)

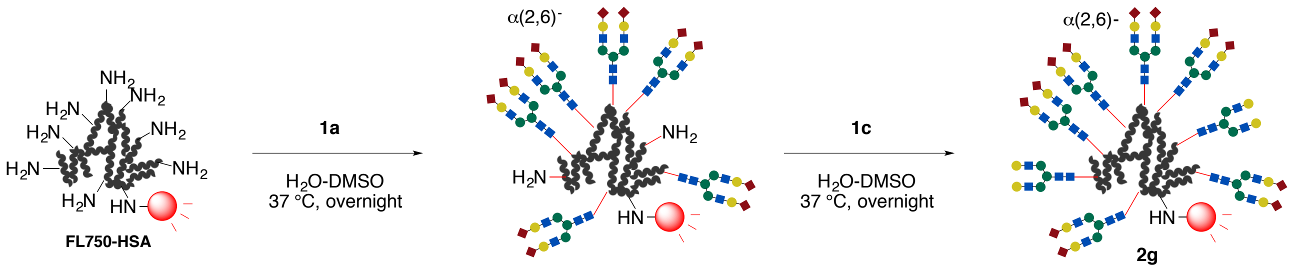

FL750-HSA stock solution prepared above (175 μL, 10 nmol) was diluted with 88 μL DMSO and 175 μL water. To this solution was added 46.7 μL stock solution of α(2,6)-disialoglycan-modifying probe **1a**, prepared above (175 nmol, 17.5 eq), and the mixture was incubated overnight at 37 °C. A small amount of the reaction mixture (0.5 μL) was analyzed by MALDI-TOF-MS (positive mode), detecting the molecular weight of the sialylated intermediate at 96.9 kDa, which contains average number, 8.3 molecules of disialoglycan **a** per albumin.

To a diluted solution of the sialylated intermediate (44 μL, 1.0 nmol) obtained above was added 2.0 μL stock solution of asialoglycan-modifying probe **1c** as prepared previously (7.5 nmol, 7.5 eq) and the mixture was incubated at 37 °C overnight. The resulting solution was diluted with water and centrifuged through Amicon 10K® at 15,000 rpm for 10 min, and further washed with water three times to filter off any small molecules. The insoluble byproducts were removed by filtering with Durapore PVDF 0.45 μm® and diluted with water to give 50 mM solution of heterogeneous glycoalbumin **2g**. MALDI-TOF-MS (positive mode) detected the molecular weight of **2g** at 103.9 kDa, which therefore contained 2.6 molecules of glycan **c** per albumin (total number of glycans introduced to albumin was 10.9).

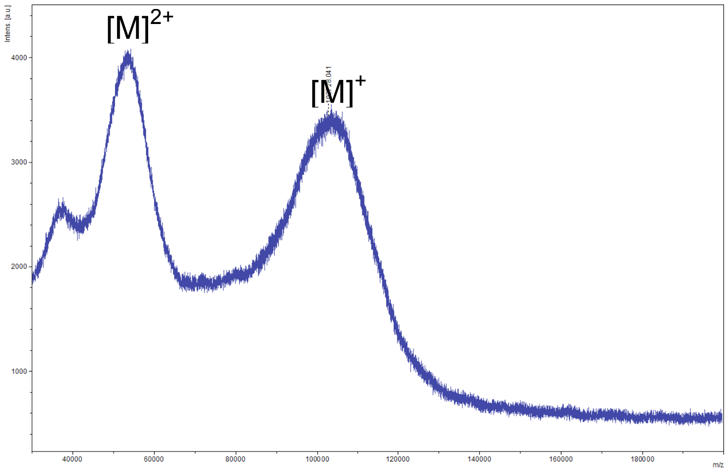

MALDI-TOF-MS of 2g

Preparation of heterogeneous glycoalbumin **2h** with α(2,6)-disialo- and asialoglycans (5 : 5)

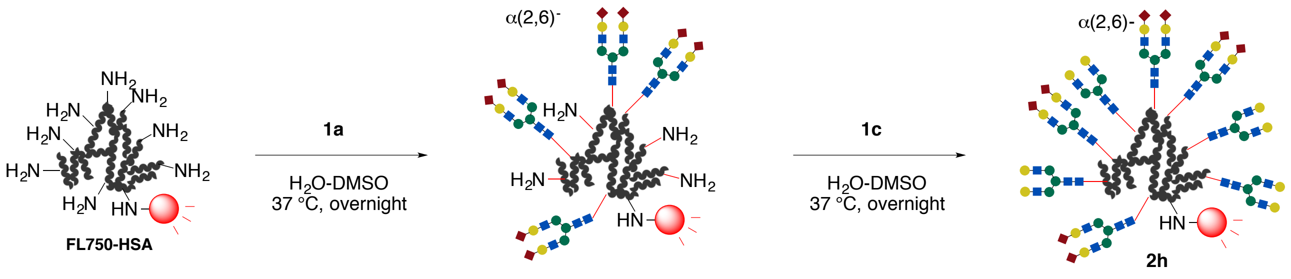

FL750-HSA stock solution prepared above (210 μL, 12 nmol) was diluted with 105 μL DMSO and 210 μL water. To this solution was added 43.4 μL stock solution of α(2,6)-disialoglycan-modifying probe **1a**, prepared above (163 nmol, 13.6 eq), and the mixture was incubated overnight at 37 °C. A small amount of the reaction mixture (0.5 μL) was analyzed by MALDI-TOF-MS (positive mode), detecting the molecular weight of the sialylated intermediate at 87.1 kDa, which contains average number, 5.3 molecules of disialoglycan **a** per albumin.

To a diluted solution of the sialylated intermediate (215 μL, 5.0 nmol) obtained above was added 14.2 μL stock solution of asialoglycan-modifying probe **1c** as prepared previously (52 nmol, 10.4 eq) and the mixture was incubated at 37 °C overnight. The resulting solution was diluted with water and centrifuged through Amicon 10K® at 15,000 rpm for 10 min, and further washed with water three times to filter off any small molecules. The insoluble byproducts were removed by filtering with Durapore PVDF 0.45 μm® and diluted with water to give 50 mM solution of heterogeneous glycoalbumin **2h**. MALDI-TOF-MS (positive mode) detected the molecular weight of **2h** at 98.7 kDa, which therefore contained 4.7 molecules of glycan **c** per albumin (total number of glycans introduced to albumin was 10.0).

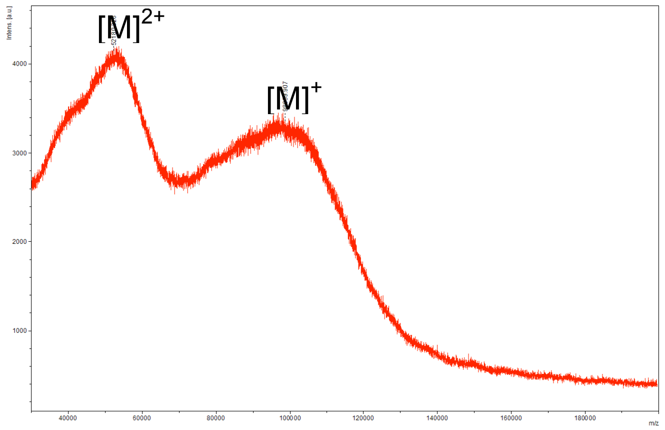

MALDI-TOF-MS of 2h

Preparation of heterogeneous glycoalbumin **2i** with α(2,6)-disialo- and asialoglycans (3 : 7)

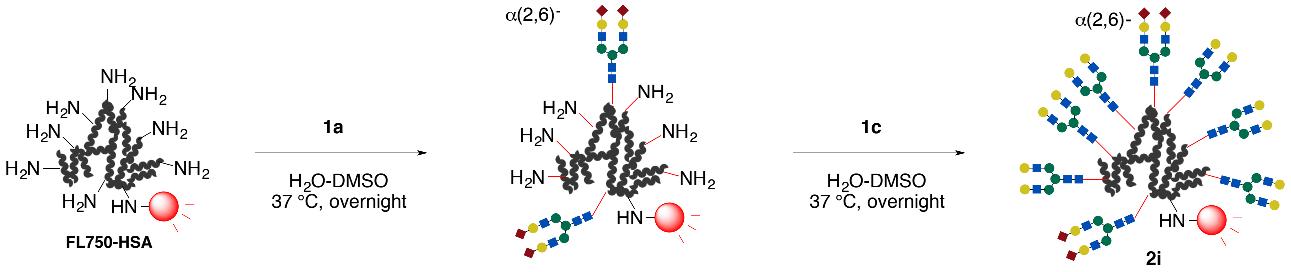

FL750-HSA stock solution prepared above (175 μL, 10 nmol) was diluted with 88 μL DMSO and 175 μL water. To this solution was added 13.3 μL stock solution of α(2,6)-disialoglycan-modifying probe **1a**, prepared above (50 nmol, 5.0 eq), and the mixture was incubated overnight at 37 °C. A small amount of the reaction mixture (0.5 μL) was analyzed by MALDI-TOF-MS (positive mode), detecting the molecular weight of the sialylated intermediate at 78.9 kDa, which contains average number, 2.8 molecules of disialoglycan **a** per albumin.

To a diluted solution of the sialylated intermediate (119 μL, 2.8 nmol) obtained above was added 15.3 μL stock solution of asialoglycan-modifying probe **1c** as prepared previously (50 nmol, 20.9 eq) and the mixture was incubated overnight at 37 °C. The resulting solution was diluted with water and centrifuged through Amicon 10K® at 15,000 rpm for 10 min, and further washed with water three times to filter off any small molecules. The insoluble byproducts were removed by filtering with Durapore PVDF 0.45 μm® and diluted with water to give 50 mM solution of heterogeneous glycoalbumin **2i**. MALDI-TOF-MS (positive mode) detected the molecular weight of **2i** at 97.2 kDa, which therefore contained 6.3 molecules of glycan **c** per albumin (total number of glycans introduced to albumin was 9.1).

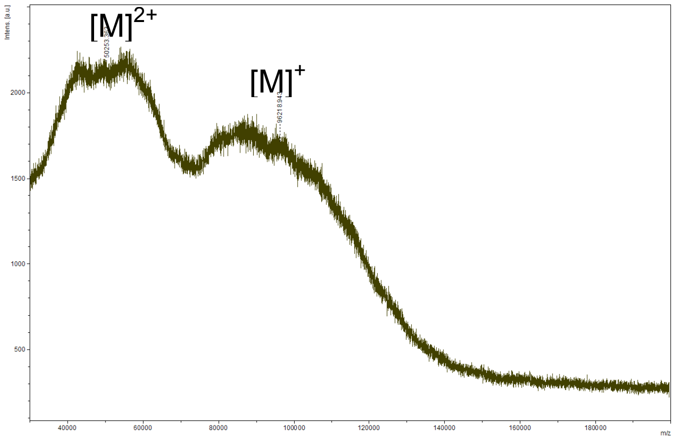

MALDI-TOF-MS of 2i

Preparation of heterogeneous glycoalbumin **2j** with asialo- and α(2,6)-disialoglycans (5 : 5, inversely modified glycoalbumin)

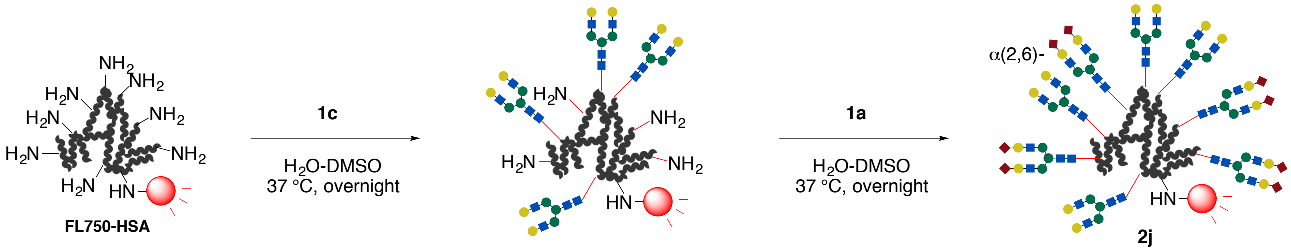

FL750-HSA stock solution prepared above (175 μL, 10 nmol) was diluted with 88 μL DMSO and 175 μL water. To this solution was added 43 μL stock solution of asialoglycan-modifying probe **1c**, prepared above (16 nmol, 16 eq), and the mixture was incubated overnight at 37 °C. A small amount of the reaction mixture (0.5 μL) was analyzed by MALDI-TOF-MS (positive mode), detecting the molecular weight of the glycosylated intermediate at 83.5 kDa, which contains average number, 5.2 molecules of asialoglycan **c** per albumin.

To a solution of the partially glycosylated intermediate (88 μL, 2.0 nmol) obtained above was added 4.3 μL stock solution of α(2,6)-disialoglycan-modifying probe **1a** as prepared previously (16 nmol, 8.0 eq) and the mixture was incubated at 37 °C overnight. The resulting solution was diluted with water and centrifuged through Amicon 10K® at 15,000 rpm for 10 min, and further washed with water three times to filter off any small molecules. The insoluble byproducts were removed by filtering with Durapore PVDF 0.45 μm® and diluted with water to give 50 mM solution of heterogeneous glycoalbumin **2j**. MALDI-TOF-MS (positive mode) detected the molecular weight of **2j** at 97.6 kDa, which therefore contained 4.7 molecules of glycan **a** per albumin (total number of glycans introduced to albumin was 9.9).

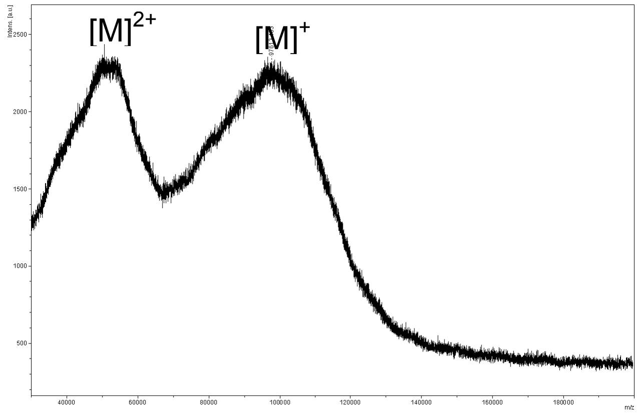

MALDI-TOF-MS of 2j

2. *In Vivo* Kinetics and biodistribution Analysis

2.1. General

1.5 nmol / 30 µL of *N*-glycoalbumins were diluted in 70 µL saline, and injected into 8 to 10-week-old BALB/cAJcl-nu/nu mice (CLEA Japan, Inc.) via the tail vein (N = 3). The mice were then anesthetized with Nembutal or isoflurane and placed in a fluorescence imager, IVIS kinetics fluorescence imager® (Caliper Life Sciences, Inc., Hopkinton, Massachusetts, USA). Abdominal and dorsal side images were taken at 30-minute intervals. To evaluate the rate of fluorescent signal increased in the urinary bladder, the fluorescence around the urinary bladder was calculated within an arbitrarily defined region of interest (ROI). After 3 hours of observation, the mice were sacrificed and perfused with 4% paraformaldehyde solution, and fluorescence intensities in the liver, spleen, gallbladder and intestine were measured within an arbitrarily defined ROI.

2.2. Supplementary images

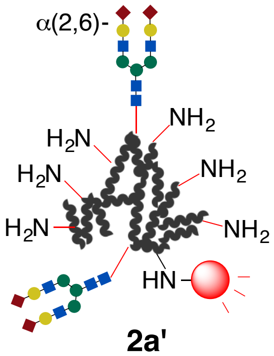

2a'

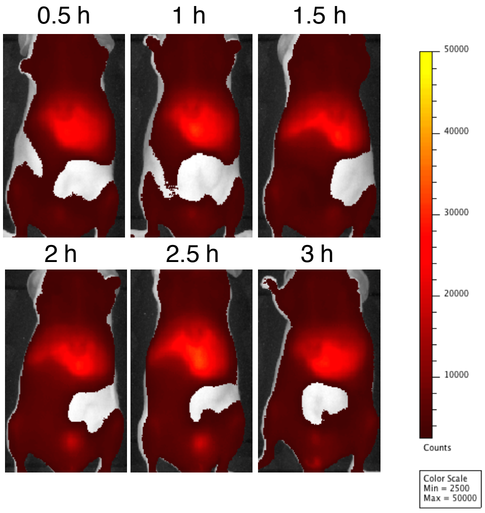

Figure S1 | *In vivo* fluorescence imaging (abdominal side) of α(2,6)-disialylated glycoalbumin **2a’** with fewer glycans. Experiments were performed as described in Figure 3.

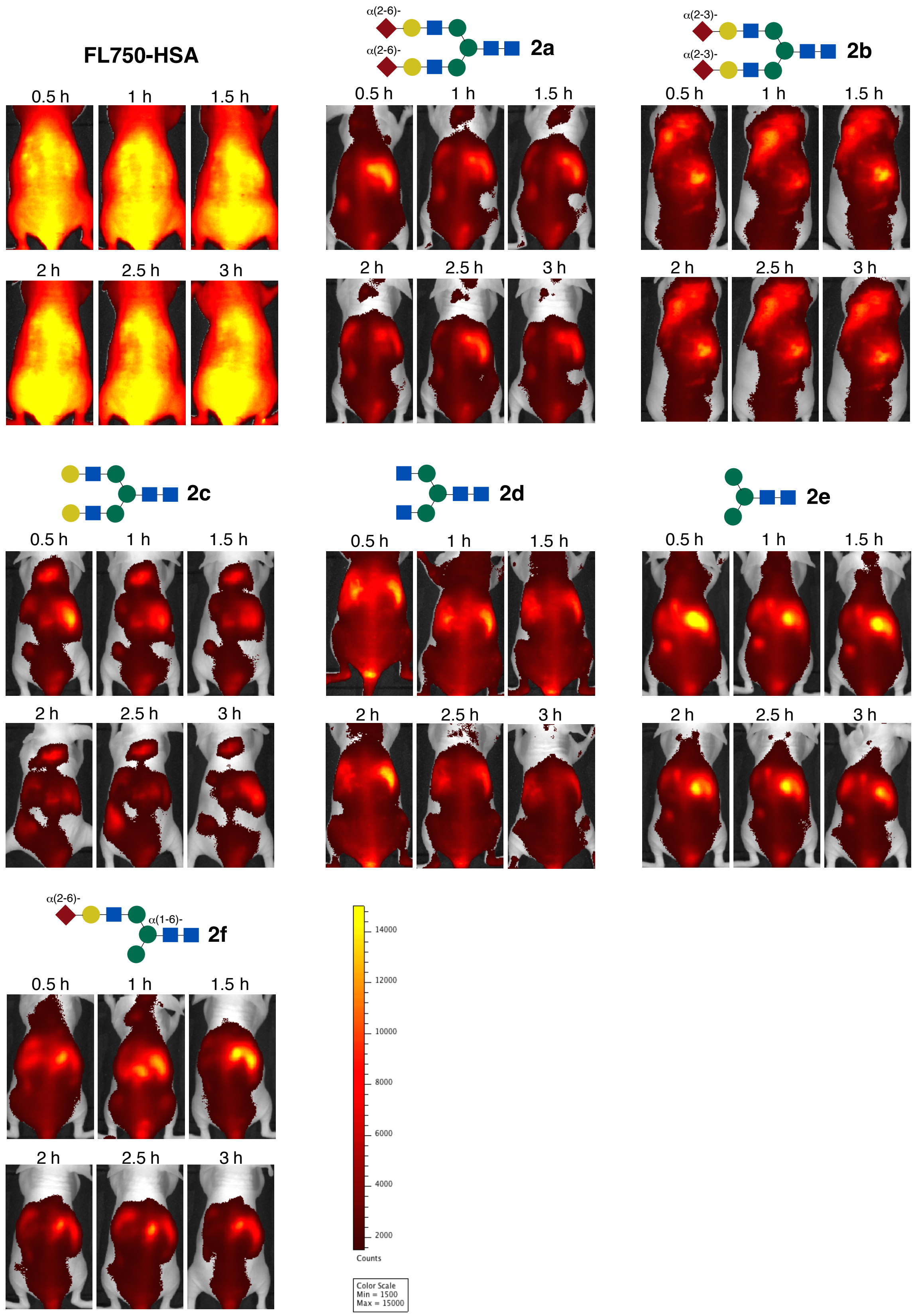

Figure S2 | *In vivo* fluorescence imaging (dorsal side) of *N*-glycoclusters **2a–f**.

Experiments were performed as described in Figure 3.

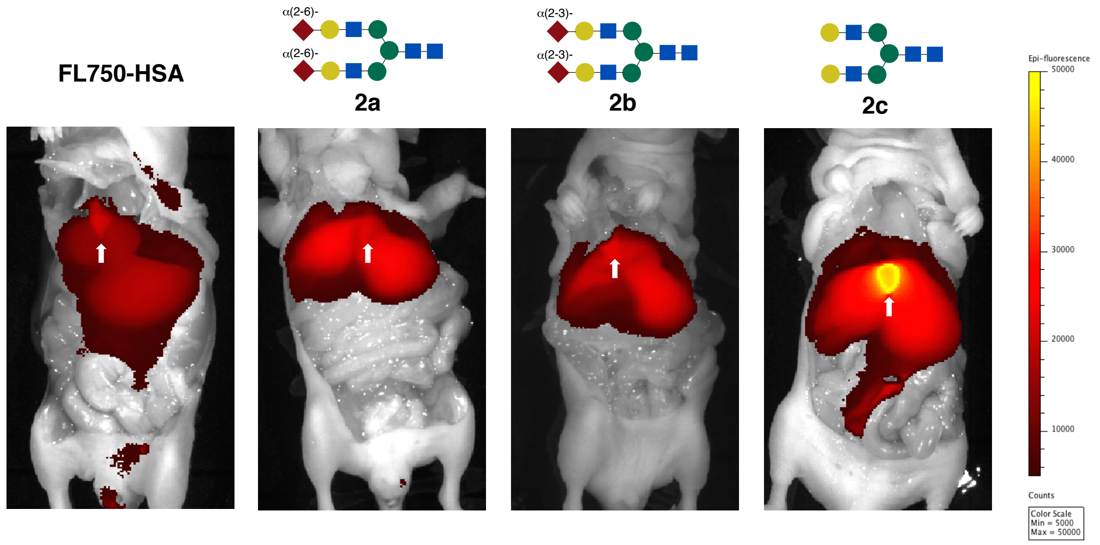

Figure S3 | Fluorescence images of mice treated with **FL750-HSA** and glycoalbumins **2a–c** after opening the abdomen (3 hours of imaging experiments). Position of gall bladder is shown by white arrow.

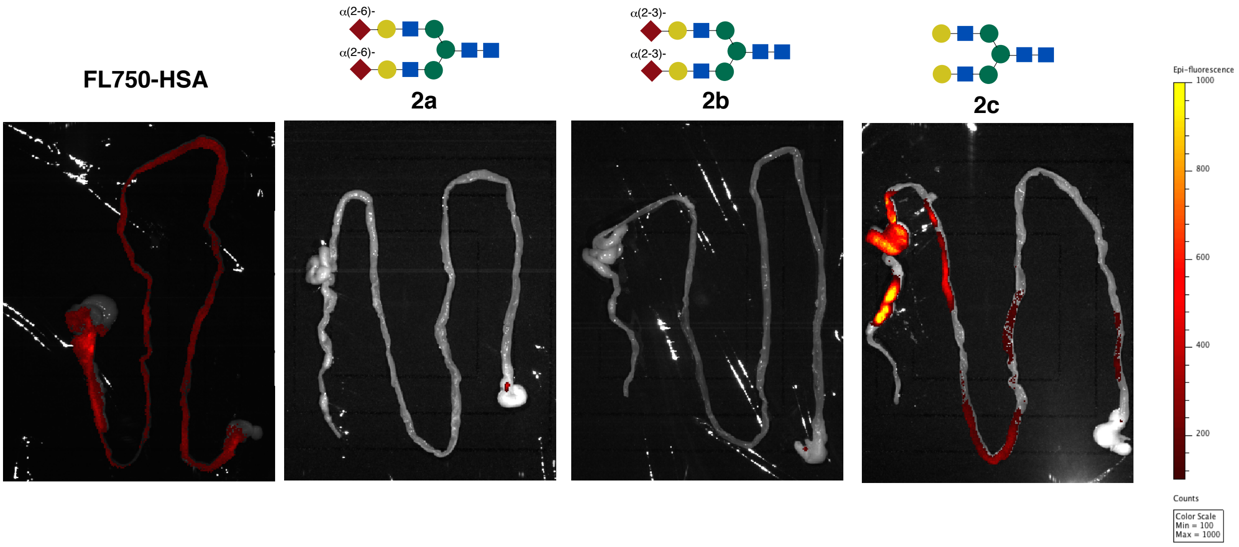

Figure S4 | Fluorescence images of isolated intestine from the mice treated with **FL750-HSA** and glycoalbumins **2a–c** after 3 hours of imaging experiments.

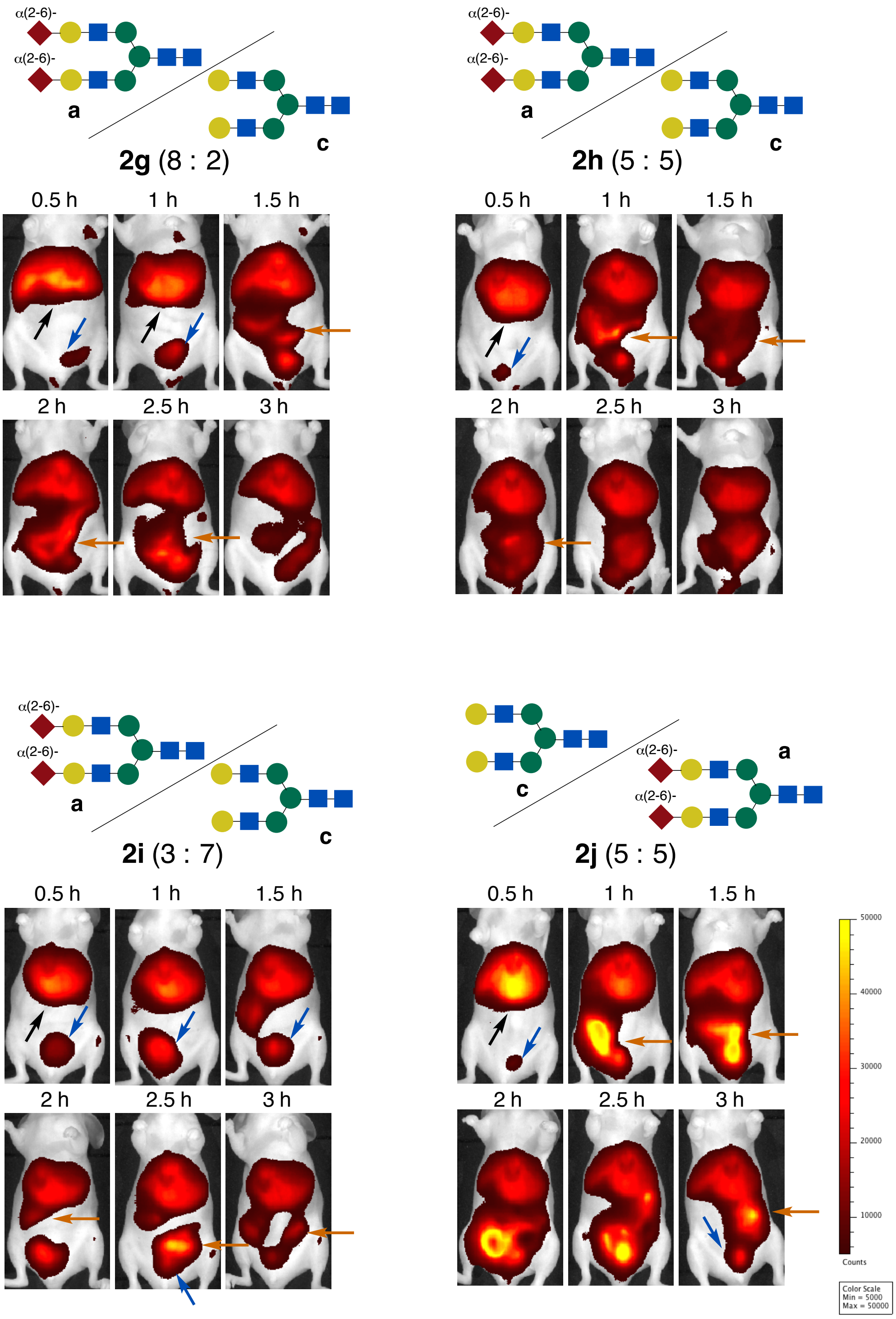

Figure S5 | *In vivo* fluorescence imaging (abdominal side) of heterogeneous *N*-glycoclusters **2g–j**. Experiments were performed as described in Figure 3. Black arrow: liver; blue arrow: excretion to urinary bladder; orange arrow: excretion to intestine.

3. Immunohistochemistry

After *in vivo* kinetics analysis described in **2.1.**, livers were further postfixed with 4% PFA at 4 °C for 24 hours, transferred to 15% sucrose in PBS and kept at 4 °C for 24 hours, then to 30% sucrose in PBS likewise. The tissues were frozen in OCT compound® at -78 °C. The tissue blocks were cut into 6-8 mm sections. After blocking with 3% BSA/10% normal goat serum/0.1 M glycine/PBST for 30 min, the sections were incubated with anti-Desmin (RB-9014, 1:300, rabbit; Thermo Fisher Scientific, Fremont, CA), anti-LYVE-1 (ab14917, 1:200, rabbit; abcam, Cambridge, UK), and anti-F4/80 (MCA497GA, 1:200, rat, AbD serotec, Oxford, UK) andbodies overnight at 4 °C, followed by incubation with both Alexa Fluor 555 anti-rat IgG (1:2000; green, Life Technologies, Carlsbad, CA) and Alexa Fluor 555 anti-rabbit IgG (1:2000; green, Life Technologies, Carlsbad, CA) for 2 hours at room temperature. Thereafter, the sections were incubated with Hoechst 33258 (1:2500); blue, DOJINDO LABORATORIES, Kumamoto, JP) for 10 min at room temperature, and were mounted with Fluoromount® (Diagnostic BioSystems, Pleasanton, CA). The slides thus prepared were placed in Keyence BZ-X710 All-in-one Fluorescence Microscope®, and observed at appropriate wavelengths.

4. References

1. Tanaka, K. et al. A cascading reaction sequence involving ligand-directed azaelectrocyclization and autooxidation-induced fluorescence recovery enables visualization of target proteins on the surface of live cells. *Org. Biomol. Chem.* **12**, 1412-1418 (2014)
2. Tanaka, K. et al. Noninvasive Imaging of Dendrimer-type N-Glycan Clusters: In Vivo Dynamics Dependence on Oligosaccharides Structure. *Angew. Chem. Int. Ed*. **49**, 8195-8200 (2010)
